# Towards apparent convergence in asymptotically safe quantum gravity

**DOI:** 10.1140/epjc/s10052-018-5806-0

**Published:** 2018-04-26

**Authors:** T. Denz, J. M. Pawlowski, M. Reichert

**Affiliations:** 1 Institut für Theoretische Physik, Universität Heidelberg, Philosophenweg 16, 69120 Heidelberg, Germany; 2 ExtreMe Matter Institute EMMI, GSI Helmholtzzentrum für Schwerionenforschung mbH, Planckstr. 1, 64291 Darmstadt, Germany

## Abstract

The asymptotic safety scenario in gravity is accessed within the systematic vertex expansion scheme for functional renormalisation group flows put forward in Christiansen et al. (Phys Lett B 728:114, 2014), Christiansen et al. (Phy Rev D 93:044036, 2016), and implemented in Christiansen et al. (Phys Rev D 92:121501, 2015) for propagators and three-point functions. In the present work this expansion scheme is extended to the dynamical graviton four-point function. For the first time, this provides us with a closed flow equation for the graviton propagator: all vertices and propagators involved are computed from their own flows. In terms of a covariant operator expansion the current approximation gives access to $\Lambda$, *R*, $R^2$ as well as $R_{\mu \nu }^2$ and higher derivative operators. We find a UV fixed point with three attractive and two repulsive directions, thus confirming previous studies on the relevance of the first three operators. In the infrared we find trajectories that correspond to classical general relativity and further show non-classical behaviour in some fluctuation couplings. We also find signatures for the apparent convergence of the systematic vertex expansion. This opens a promising path towards establishing asymptotically safe gravity in terms of apparent convergence.

## Introduction

A consistent formulation of quantum gravity (QG) is an open problem, with many contenders being investigated in great detail. In the past two decades, Weinberg’s asymptotic safety scenario (AS) proposed in 1976 [4] has been investigated with the help of non-perturbative renormalisation group techniques. AS posits that QG, while being perturbatively non-renormalisable, is non-perturbatively renormalisable and features a non-trivial fixed point in the ultraviolet (UV).

By now an impressive body of evidence has been collected that supports this intriguing scenario: the application of functional renormalisation group (FRG) techniques [5] to QG [6] has allowed for a confirmation of the existence of the non-trivial UV fixed point in basic Einstein-Hilbert approximations [6–8]. Later works have improved on these approximations and gone beyond Einstein-Hilbert [1–3, 9–52]; for an overview see reviews [53–56]. Furthermore, the stability of the asymptotic safety setting for gravity-matter systems, [57–59, 61–76], as well as asymptotically safe theories within a perturbative setup, [77–82], have also attracted a lot of attention.

In [3] the systematic vertex expansion in quantum gravity initiated in [1, 2], see also [26, 33], has been pushed to the graviton three-point function, for the first time including a dynamical graviton-scattering in the asymptotic safety analysis. Here we extend the vertex expansion to the graviton four-point function. Apart from the significant technical challenge such an upgrade of the approximation has posed, we think that this constitutes a necessary and significant progress towards asymptotically safe gravity:

- As such it is an important step towards apparent convergence of the vertex expansion in quantum gravity: apparent convergence aims at the convergence of vertices as well as observables in the order of a given systematic expansion scheme; here we use the vertex expansion scheme. Together with the investigation of the regulator (in-)dependence of observables this provides a systematic error estimate in the present approach, and should be compared with apparent continuum scaling and extrapolation on the lattice.
- The present approximation allows for the identification of diffeomorphism-invariant structures in the vertex expansion, i.e. $R^2$ and $R_{\mu \nu }^2$ tensor structures as well as those of higher derivative invariants. This is not only important for getting access to the number of relevant directions at the asymptotically safe UV fixed point in quantum gravity, but can also provide non-trivial support and additional information for computations within the standard background field approximation.
- It is the first approximation in the asymptotic safety approach to gravity where the flow of the pivotal building block, the two-point correlation function or (inverse) propagator, is closed: The flows of all involved vertex functions are computed within given approximations. As the propagator is the core object in the present approach, we consider this an important milestone on the way towards asymptotically safe quantum gravity.

As a main result of this paper, we find further significant evidence for a non-trivial UV fixed point in quantum gravity. This fixed point has three relevant directions and two repulsive ones. The three relevant directions can be associated with the cosmological constant (graviton mass parameter), Newton’s coupling and the $R^2$-coupling, see Sect. 5. We also investigate the stability of this UV fixed point and observe that the system is significantly less sensitive to the closure of the flow equations than previous truncations. In addition, we observe that the critical exponents also become less sensitive to the details of the approximations. These are two necessary signatures of apparent convergence. Furthermore, we investigate the infrared (IR) behaviour of the system and find trajectories connecting the UV fixed point with classical general relativity in the IR.

This work is structured in the following way: In Sect. 2 we elaborate on the important property of background independence of quantum gravity and its consequences for the dynamical correlation functions. In Sect. 3, we briefly introduce the functional renormalisation group and the covariant vertex expansion used in the present work. Next, Sect. 4 presents our setup and the derivations of the flow equations for the couplings. We especially focus on how the tensor structures of higher curvature invariants are embedded in our vertex expansion. In Sect. 5, we present our results, in particular the non-trivial UV fixed point with three attractive and two repulsive directions. Furthermore, we test the stability of the UV fixed point with respect to change of the precise truncation, and find that in almost all approximations we obtain a similar UV fixed point. In Sect. 6, we investigate the IR-behaviour of the UV-finite trajectories. Our analysis confirms the classical IR fixed points found before in the vertex expansion. In Sect. 7 we discuss the convergence of the present vertex expansion scheme with an increasing number of flowing *n*-point correlation functions. Finally, in Sect. 8 we summarise our results.

## Diffeomorphism invariance and background independence

The quantum field theoretical formulation of quantum gravity in terms of metric correlation functions necessitates the introduction of a background metric ${\bar{g}}$, and quantum fluctuations are taken to be fluctuations about this background metric. This begs the question of whether diffeomorphism invariance and background independence of observables are guaranteed in such a framework. While this is an important question, its answer is not directly relevant for the computations presented here. Hence, the present chapter may be skipped in a first reading.

In the present work we perform computations in the linear split, where the full metric *g* is given by $g={\bar{g}}+h$. More general splits, $g={\bar{g}}+f({\bar{g}},h)$ have been considered for example within the geometrical or Vilkovisky-deWitt approach, e.g. [26, 83–85], or the exponential split, e.g. [40, 44, 68, 86–88]. Observables, on the other hand, are background independent. This property is encoded in the Nielsen (NI) or split-Ward identities (SWI) that relate derivatives of the effective action $\Gamma [\bar{g},\phi ]$ w.r.t. the background metric ${\bar{g}}$ to those w.r.t. the graviton fluctuations *h*. Here we have introduced the fluctuation superfield

1 $$\begin{aligned} \phi =(h,c,{\bar{c}}). \end{aligned}$$

The (anti-) ghost fields, *c* and ${\bar{c}}$, stem from the Faddeev-Popov gauge fixing procedure, see next section. The effective action generates all one-particle-irreducible correlation functions and as such encodes the symmetries of the theory. Schematically, these identities read [6, 20, 26, 84, 89–95]

2 $$\begin{aligned} \frac{\delta \Gamma [{\bar{g}},h]}{\delta {\bar{g}}(x)} = \int _{y}{\mathcal C}[{\bar{g}}, h](x,y)\frac{\delta \Gamma [{\bar{g}}, h]}{\delta h(y)}+{\mathcal N}[{\bar{g}}, h](x), \end{aligned}$$

where we have suppressed the ghost fields. In the linear split we have ${\mathcal C}[{\bar{g}}, h](x,y)=\delta (x-y)$ and the second term ${\mathcal N}[{\bar{g}}, h]$ carries the information about the non-trivial behaviour under diffeomorphism transformations of the gauge fixing sector and the regularisation. In turn, in the geometrical approach diffeomorphism invariance of the effective action is achieved by a non-linear split with $f({\bar{g}},h)$ leading to the non-trivial prefactor ${\mathcal C}[{\bar{g}}, h]$ in (2). The term ${\mathcal N}[{\bar{g}}, h]$ then carries the deformation of the Nielsen identity in the presence of a regularisation but does not spoil diffeomorphism invariance.

In both cases the Nielsen identity is a combination of a quantum equation of motion, the Dyson-Schwinger equation, and the Slavnov-Taylor identity (STI) or diffeomorphism constraint. The setup also entails that correlations of the fluctuation fields are necessarily background-dependent. This is easily seen by iterating (2). Moreover, in the linear split, diffeomorphism invariance of the observables is encoded in non-trivial STIs for the fluctuation correlation functions, while in the geometrical formulation, the non-trivial STIs are encoded in expectation values of $f({\bar{g}},h)$ and its derivatives.

Due to (2) we have to deal with the peculiarity that background independence and physical diffeomorphism invariance of observables necessitate background-dependence and non-trivial STIs for the correlation functions of the fluctuation fields. This leads to seemingly self-contradictory statements: in particular, for the quantum effective action $\Gamma [{\bar{g}}, h]$ it entails that physical diffeomorphism invariance of observables is not achieved by diffeomorphism invariance w.r.t. diffeomorphism transformations of the fluctuation fields. The latter does not do justice to either diffeomorphism invariance or background independence.

This peculiarity can easily be checked in a non-Abelian gauge theory within the background field formulation: in a fluctuation gauge invariant approximation to the effective action, even two-loop universal observables such as the two-loop $\beta$-function cannot be computed correctly. Indeed, in this case it is well-known that only the non-trivial STIs for the fluctuation gauge field elevate the auxiliary background gauge invariance to the physical one holding for observables, see e.g. [96, 97].

The above considerations underline the importance of a direct computation of correlation functions of the fluctuation field *h*. Indeed, the corresponding set of flow equations for $\Gamma ^{(n)}=\Gamma ^{(0,n)}$ is closed in the sense that the flow diagrams only depend on $\Gamma ^{(n)}$ with $n\ge 2$. Here, $\Gamma ^{(n,m)}$ stands for the *n*-th background field derivative and *m*-th fluctuation field derivative of the effective action,

3 $$\begin{aligned} \Gamma ^{(n,m)}[{\bar{g}},\phi ] :=\frac{\delta ^{n+m}\Gamma [{\bar{g}},h]}{\delta {\bar{g}}^n \delta \phi ^m}. \end{aligned}$$

In turn, the flows for pure background, $\Gamma ^{(n,0)}$, or mixed background-fluctuation functions, $\Gamma ^{(n,m)}$ with $m\ne 0$, necessitate the fluctuation correlation functions as an input: the background correlation functions can be iteratively computed in powers of the background metric. In other words, the dynamics of the system is solely determined by the pure fluctuation correlation functions.

For this reason, several approaches for computing correlation functions of the fluctuation field have been put forward in the last years. Some of these approaches are set up for computing both correlation functions of the fluctuation field as well as those of the background field: vertex expansion [1–3, 33, 71, 73, 74] and bimetric approach [20, 24, 37, 90]. Another set of approaches relies on utilising the Nielsen or split Ward identities explicitly or implicitly, [26, 84, 92–95, 98–102].

So far, the $\Gamma ^{(n)}$ for $n\ge 2$ have been computed directly in only one approach, the vertex expansion, see [1–3, 33] for pure gravity and [71, 73, 74] for gravity-matter systems. A mixture of vertex expansion and background approximation has been used in [33, 67, 72]. Present results include $\Gamma ^{(n)}$ for $n=0,1,2,3$, where higher vertices have been estimated by lower ones, relying on approximate covariance of the correlation functions. Such a structure has already been confirmed in the perturbative and semi-perturbative regime of QCD, see [103].

## Effective action and functional renormalisation group

The set of (covariant) correlation functions of the metric, $\langle g(x_1)\cdots g(x_n)\rangle$, defines a given theory of quantum gravity. All observables can be constructed from these basic building blocks. The correlation functions are generated from the single metric effective action, $\Gamma [g]=\Gamma [g, h=0]$, which is the free energy in a given metric background $g={\bar{g}} +h$ at $h=0$. Here we have restricted ourselves to a linear split. The underlying classical action is the Einstein-Hilbert action,

4 $$\begin{aligned} S_\text {EH}&= \frac{1}{16 \pi G_N} \int \mathop {\mathrm {d}^{4} x} \sqrt{\det g} \Bigl (2\Lambda -R(g)\Bigr ) + S_\text {gf}+ S_\text {gh}, \end{aligned}$$

where $R(g)$ is the Ricci curvature scalar, while $S_\text {gf}[\bar{g},h]$ and $S_\text {gh}[{\bar{g}}, \phi ]$ describe the gauge-fixing and Faddeev-Popov ghost parts of the action, respectively. The gauge fixing action reads

5 $$\begin{aligned} S_\text {gf}[{\bar{g}},h]=\frac{1}{2\alpha }\int \mathrm d^4x\, \sqrt{\det \bar{g}}\,{\bar{g}}^{\mu \nu }F_\mu F_\nu . \end{aligned}$$

We employ a linear, de-Donder type gauge-fixing,

6 $$\begin{aligned} F_\mu&= {\bar{\nabla }}^\nu h_{\mu \nu } - \frac{1+\beta }{4} {\bar{\nabla }}_\mu {h^\nu }_\nu . \end{aligned}$$

In particular we use the harmonic gauge given by $\beta =1$. This choice simplifies computations considerably due to the fact that the poles of all modes of the classical graviton propagator coincide. We furthermore work in the limit of a vanishing gauge parameter, $\alpha \rightarrow 0$. This is favourable because then the gauge does not change during the flow since $\alpha =0$ is a RG fixed point [104]. Moreover, it allows for a clear separation of propagating and non-propagating degrees of freedom. The ghost part of the action reads

7 $$\begin{aligned} S_\text {gh}[{\bar{g}},\phi ]=\int \mathrm d^4x \,\sqrt{\det \bar{g}}\, \bar{c}^\mu \mathcal {M}_{\mu \nu } c^\nu \,, \end{aligned}$$

where ${\bar{c}}$ and *c* denote the (anti-) ghost field and $\mathcal {M}$ is the Faddeev-Popov operator deduced from (6). For $\beta =1$ it is given by

8 $$\begin{aligned} \mathcal {M}_{\mu \nu }&= \bar{\nabla }^\rho \left( g_{\mu \nu }\nabla _\rho +g_{\rho \nu }\nabla _\mu \right) -\bar{\nabla }_\mu \nabla _\nu \,. \end{aligned}$$

The gauge fixing and ghost term in (5) and (7) introduce the separate dependence on ${\bar{g}}$ and *h* leading to the non-trivial Nielsen identities in (2).

### Flow equation

An efficient way of computing non-perturbative correlation functions is the functional renormalisation group. In its form for the effective action, see [5, 105, 106], it has been applied to quantum gravity [6]. For reviews on the FRG approach to gauge theories and gravity see e.g. [53–56, 89, 107]. The RG flow of the effective action for pure quantum gravity is given by

9 $$\begin{aligned} {\partial _t \Gamma }_k&= \frac{1}{2} {{\mathrm{Tr}}}\left[ \frac{1}{\Gamma _{k}^{(2)}+R_{k}} \partial _t R_k \right] _{hh} - {{\mathrm{Tr}}}\left[ \frac{1}{\Gamma _{k}^{(2)}+R_{k}} \partial _t R_k \right] _{\bar{c}c}. \end{aligned}$$

Here, $\partial _t \equiv k \partial _k$ denotes the scale derivative, where *k* is the infrared cutoff scale. $\Gamma _{k}^{(2)}=\Gamma _k^{(0,2)}$ stands for the second fluctuation field derivative of the effective action, while $R_k$ is the regulator, which suppresses momenta below *k*. The trace in (9) sums over internal indices and integrates over space-time.

The introduction of cutoff terms leads to regulator-dependent modifications of STIs and NIs that vanish for $R_k\rightarrow 0$. The respective symmetry identities have hence been named modified Slavnov-Taylor identities (mSTIs) and modified Nielsen- or split Ward identities (mNIs/mSWIs). The modification entails the breaking of the physical or quantum diffeomorphism invariance in the presence of a background covariant momentum cutoff. Still, background diffeomorphism invariance is maintained in the presence of the cutoff term.

### Covariant expansion

The effective action $\Gamma _{k} [\bar{{g}}, \phi ]$ depends on the background metric $\bar{{g}}$ and the fluctuation superfield $\phi =(h,c,{\bar{c}})$, see (1), separately. The functional flow equation (9) is accompanied by the functional mSTIs & mNIs for the effective action that monitor the breaking of quantum diffeomorphism invariance, see (2) in Sect. 2.

In order to solve (9), we employ a vertex expansion around a given background $\bar{{g}}$, to wit

10 $$\begin{aligned} \Gamma _k\left[ \bar{{g}},\phi \right] = \sum _{n=0}^{\infty } \frac{1}{n !} \Gamma _k^{(\phi _{i_1}\ldots \phi _{i_n})}\left[ \bar{{g}},0\right] \, \prod _{l=1}^n \phi _{i_l}, \end{aligned}$$

where the superscript fields in parentheses are a short-hand notation for field derivatives, and where contracting over super-indices $i_j$ occurring twice is implied. In this work, we include the full flow of the vertex functions up to the graviton four-point function.

As discussed in Sect. 2, the expansion coefficients $\Gamma _k^{(\phi _{i_1}\ldots \phi _{i_n})}=\Gamma _k^{(n)}=\Gamma _k^{(0,n)}$ satisfy mSTIs as well as mNIs with $\Gamma ^{(n,m)}_k$ being defined in (3). For the sake of simplicity we now restrict ourselves to the gauge fixing used in the present work, (6) with $\alpha =0$. Then the fluctuation graviton propagator is transverse: it is annihilated by the gauge fixing condition.

An important feature of the functional RG equations is that for $\alpha =0$ the flow equations for the transverse vertices $\Gamma ^{(n)}_{k,T}$ are closed: the external legs of the vertices in the flow are transverse due to the transverse projection of the flow, the internal legs are transverse as they are contracted with the transverse propagator. Schematically this reads

11 $$\begin{aligned} \partial _t \Gamma ^{(n)}_{k,T}={\text {Flow}}^{(n)}_T[\{\Gamma ^{(m)}_{k,T}\}]. \end{aligned}$$

In other words, the system of transverse fluctuation correlation functions is closed and determines the dynamics of the system. On the other hand, the mSTIs are non-trivial relations for the longitudinal parts of vertices in terms of transverse vertices and longitudinal ones. This leads us to the schematic relation

12 $$\begin{aligned} \Gamma ^{(n)}_{k,L}=\mathrm {mSTI}^{(n)}[\{\Gamma ^{(m)}_{k,T}\}, \{\Gamma _{k,L}^{(m)}\}], \end{aligned}$$

see [108] for non-Abelian gauge theories. In consequence, the mSTIs provide no direct information about the transverse correlation functions without further constraint. In the perturbative regime this additional constraint is given by the uniformity of the vertices, for a detailed discussion in non-Abelian gauge theories see [109].

Accordingly, our task reduces to the evaluation of the coupled set of flow equations for the transverse vertices $\Gamma _{k,T }^{(n)}$. Each transverse vertex can be parameterised by a set of diffeomorphism-invariant expressions. Restricting ourselves to local invariants and second order in the curvature we are left with

13 $$\begin{aligned} R\,,\quad R^2\,,\quad R_{\mu \nu }^2. \end{aligned}$$

The square of the Weyl tensor $C^2$ is eliminated via the Gauß-Bonnet term, which is a topological invariant. Higher-derivative terms, such as

14 $$\begin{aligned} R^{\mu \nu } f_{\mu \nu \rho \sigma }(\nabla )R^{\rho \sigma }\qquad \text {with}\qquad f(0)= 0, \end{aligned}$$

are also taken into account. Without the constraint $f(0)=0$, Eq. (14) also includes $R^2$ and $R_{\mu \nu }^2$, more details on this basis can be found in Sect. 4. Note that also non-diffeomorphism-invariant terms are generated by the flow. In Sect. 4.3 we discuss all invariants which are included in the parameterisation of our vertices.

For the background vertices $\Gamma _k^{(n,0)}$ we use the following: the NIs become trivial in the IR as we approach classical gravity, as shown in Sect. 6. Moreover, for one of the two IR fixed points this implies that the derivative with respect to a background field is the same as a derivative with respect to a fluctuation field. This allows us to impose the trivial NIs in the IR, and all couplings are related. Then, the couplings at $k> 0$ follow from the flow equation. However, for the fluctuation couplings this amounts to solving a fine-tuning problem in the UV, for more details see Sect. 6. The latter is deferred to future work.

## Flows of correlation functions

In this chapter we discuss the technical details of the covariant expansion scheme used in the present work, including the approximations used and their legitimisation. In our opinion, a careful reading of this chapter is essential for a full understanding of the results obtained in the present work. This applies in particular to Sect. 4.1.

### Covariant tensors and uniformity

**Fig. 1:**
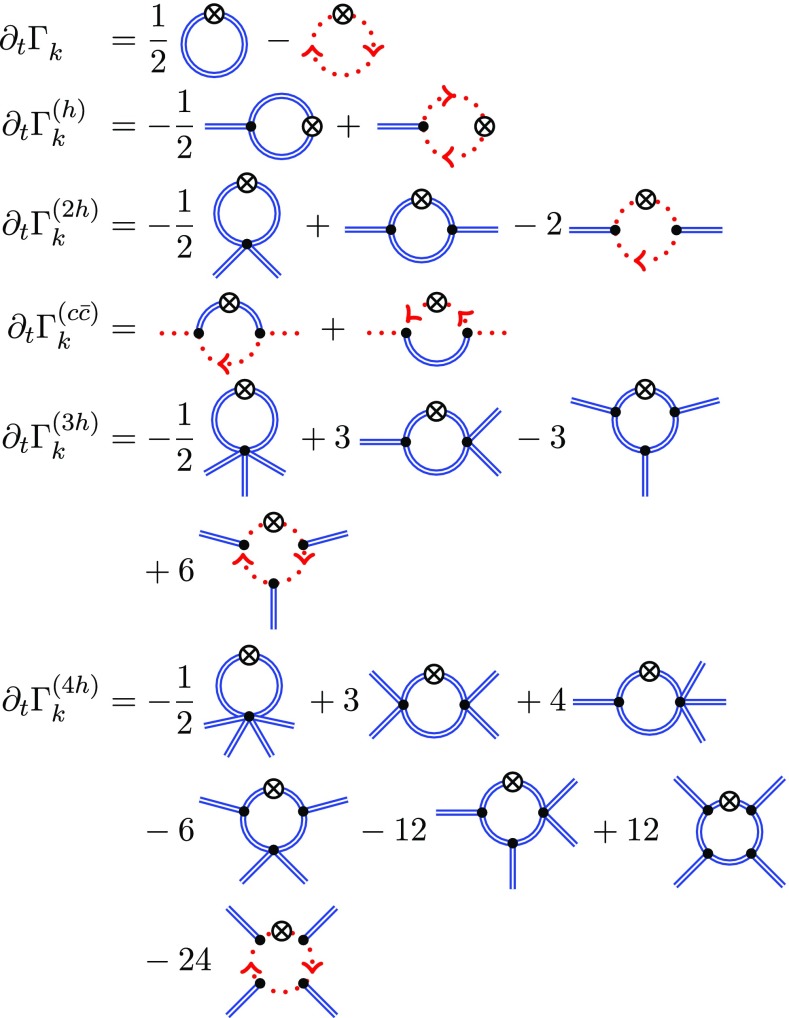
Diagrammatic representation of the flow of the vertex functions up to the graviton four-point function. The flow of any *n*-point function depends on the $(n+1)$- and $(n+2)$-point functions. Double and dotted lines represent graviton and ghost propagators, respectively. All vertices are dressed and denoted by filled circles. Crossed circles stand for regulator insertions. Symmetrisation with respect to interchange of external momenta $p_i$ is understood

The flows of the *n*-point correlation functions are generated from the FRG Eq. (9) by taking *n*-th order fluctuation field derivatives in a background ${\bar{g}}$, (see Fig. 1). In order to solve the flow equation, we employ a vertex ansatz [1, 110] including the flow of all relevant vertices up to the graviton four-point function. This vertex ansatz disentangles the couplings of background and fluctuation fields by introducing individual couplings $\Lambda _{n}$ and $G_{n}$ for each $n$-point function. These individual couplings are introduced at the level of the *n*-point correlators and replace the cosmological constant $\Lambda$ and Newton’s coupling $G_N$ of the classical Einstein-Hilbert action after performing the respective field derivatives. In summary, for the flat background $\bar{g}=\delta$ our vertex ansatz reads

15 $$\begin{aligned} \Gamma _{k}^{(\phi _1 ...\phi _n)}(\varvec{p}) = \left( \prod _{i=1}^n Z^{\frac{1}{2}}_{\phi _i} (p_i^2)\right) G_{n}^{\frac{n}{2}-1}(\varvec{p}) \mathcal {T}^{(\phi _1 ...\phi _n)}(\varvec{p};\Lambda _{n}), \end{aligned}$$

where

16 $$\begin{aligned} \mathcal {T}^{(\phi _1 ...\phi _n)}(\varvec{p};\Lambda _{n})&= G_N\,S_\text {EH}^{(\phi _1 ...\phi _n)}(\varvec{p};\Lambda \rightarrow \Lambda _{n}), \end{aligned}$$

denote the tensor structures extracted from the classical gauge-fixed Einstein-Hilbert action (4). The only flowing parameter in these tensors $\mathcal {T}^{(\phi _1 ...\phi _n)}$ is $\Lambda _n$, while $G_n(\varvec{p})$ carries the global scale- and momentum dependence of the vertex. In the above equations, $\varvec{p} = (p_{\phi _1},...,p_{\phi _n})$ denotes the momenta of the external fields $\phi _i$ of the vertex.

Apart from their flow equations, the *n*-point functions in (15) also satisfy standard RG-equations, see e.g. [89]. These RG-equations entail the reparameterisation invariance of the theory under a complete rescaling of all scales including *k*. With the parameterisation given in (15), this RG-running is completely carried by the wave function renormalisations $Z^{}_{\phi _i} (p_i^2)$ of the fields $\phi _i$, see e.g. [2, 71, 110]. Consequently, the $G_n$ and $\Lambda _n$ are RG-invariant, and hence are more directly related to observables such as *S*-matrix elements. This parameterisation of the vertices also ensures that the wave function renormalisations never appear directly in the flow equations, but only via the anomalous dimensions

17 $$\begin{aligned} \eta _{\phi _i} (p_i^2) :=- \partial _t \ln Z_{\phi _i}(p_i^2)\,. \end{aligned}$$

$G_{n}(\varvec{p})$ is the gravitational coupling of the *n*-point function, while $\Lambda _{n}$ denotes the momentum-independent part of the correlation function. In particular, $\Lambda _{2}$ is related to the graviton mass parameter $M^2 :=-2 \Lambda _{2}$. Finally, all the parameters $Z_{\phi _i}$, $G_{n}$, and $\Lambda _{n}$ are scale-dependent, but we have dropped the subscript *k* in order to improve readability.

In principle, all tensor structures, including non-diffeomorphism-invariant ones, are generated by the flow, but for our vertex functions we choose to concentrate on the classical Einstein-Hilbert tensor structures in the presence of a non-vanishing cosmological constant. Despite the restriction to these tensor structures, the $n$-point functions have an overlap with higher curvature invariants via the momentum dependence of the gravitational couplings. For example, the complete set of invariants that span the graviton wave function renormalisation is given by

18 $$\begin{aligned} R\,,\qquad R^{\mu \nu } f^{(2)}_{\mu \nu \rho \sigma }(\nabla ) R^{\rho \sigma }, \end{aligned}$$

where the superscript indicates that is a covariant tensor contributing to the two-point correlation function. Note also that we now drop the restriction on *f* present in (14). Then, this invariant naturally includes $R^2$ and $R_{\mu \nu }^2$ as the lowest order local terms. If we also allow for general momentum-dependencies, the corresponding covariant functions *f* are given by given by

19 $$\begin{aligned} f^{(2)}_{ R^2,\mu \nu \rho \sigma } =&\,\delta _{\mu \nu }\delta _{\rho \sigma } P^{(2)}_{R^2}(-\nabla ^2),\nonumber \\ f^{(2)}_{R_{\mu \nu }^2,\mu \nu \rho \sigma } =&\,\frac{1}{2} \left( \delta _{\mu \rho }\delta _{\nu \sigma }+\delta _{\mu \sigma }\delta _{\nu \rho }\right) P^{(2)}_{R_{\mu \nu }^2}(-\nabla ^2). \end{aligned}$$

The lowest order local terms, $R^2$ and $R_{\mu \nu }^2$, are given by $P^{(2)}_{R^2}=1$ and $P^{(2)}_{R_{\mu \nu }^2}=1$, respectively. Note that (19) also allows for non-local terms in the IR, i.e. anomaly-driven terms with $P^{(2)}_{R^2}=1/\nabla ^2$, see e.g. [111]. In turn, higher curvature invariants do not belong to the set of the graviton wave function renormalisation since they are at least cubic in the graviton fluctuation field.

In the present work we resort to a uniform graviton propagator in order to limit the already large computer-algebraic effort involved. The uniform wave function renormalisation is then set to be that of the combinatorially dominant tensor structure, the transverse-traceless graviton wave function renormalisation, thereby estimating the wave function renormalisations of the other modes by the transverse-traceless one. Such uniform approximations have been very successfully used in thermal field theory. There, usually the tensor structures transverse to the heat-bath are used as the uniform tensor structure, for a detailed discussion see e.g. [112] and references therein. This approximation is typically supported by combinatorial dominance of this tensor structure in the flow diagrams. Indeed, as already indicated above, the transverse-traceless mode gives the combinatorially largest contribution to the flow of the vertices computed here. Note that such an approximation would get further support if the *R* tensor structures dominate the flows, which indeed happens in the present computation.

Within this approximation the $R^2$ tensor structures drop out on the left-hand side of the graviton flow, since $R$ is already quadratic in the transverse-traceless graviton fluctuation field: in other words, the tensors defined by $f^{(2)}_{R^2}$ in (19) have no overlap with the transverse-traceless graviton.

The set of invariants that span the gravitational coupling $G_{3}(\varvec{p})$ is given by

20 $$\begin{aligned} R\,, \qquad R^{\mu \nu } f^{(3)}_{\mu \nu \rho \sigma }(\nabla ) R^{\rho \sigma }, \qquad R^{\mu \nu } R^{\rho \sigma } f^{(3)}_{\mu \nu \rho \sigma \omega \zeta }(\nabla ) R^{\omega \zeta }. \end{aligned}$$

Again, the invariants $R^2$ and $R^3$ can be excluded from this set due to their order in transverse-traceless graviton fluctuation fields. In consequence, $G_{4}(\varvec{p})$ is the only coupling in our setup that has overlap with $R^2$ contributions and higher terms in $f^{(4)}_{R^2}$.

Furthermore, in Sect. 4.3 we show that the by far dominant contribution to $G_{3}(\varvec{p})$ in the momentum range $0\le p^2\le k^2$ stems from the invariant $R$. All higher momentum dependencies of the graviton three-point function are covered by the momentum dependence of the graviton wave function renormalisation. This was already observed in [3]. As already briefly mentioned above, it gives further support to the current uniform approximation: the assumption of uniformity allows us to restrict ourselves to computing the Einstein-Hilbert tensor structure for the transverse-traceless graviton as the combinatorially dominating tensor structure. The striking momentum-independence of the actual numerical flows supports a momentum-independent approximation of $G_{3}$. In terms of (19) it implies that the dominant tensor structure for the transverse-traceless mode is given by $f^{(3)}_R$ with $P^{(3)}_R=1$. The $R_{\mu \nu }^2$ tensor structure vanishes approximately, see (27).

In contrast to the situation for the two- and three point function, the $R^2$ invariant overlaps with our transverse-traceless projection for the graviton four-point function. Indeed, its flow receives significant contributions from the invariant $R^2$. It follows that for the graviton four-point function $R$ is not the only dominant invariant in the momentum range from $p=0$ to $p=k$, as we show in Sect. 4.3. In consequence we either have to disentangle contributions from $R$ and $R^2$ tensor structures in terms of an additional tensor structure or we resolve the momentum dependence of $G_{4}(\varvec{p})$. In the present work we follow the latter procedure, see Sect. 4.5 for details.

### Projection onto $n$-point functions

The flow equations for the couplings $\Lambda _{n}$ and $G_{n}$ are obtained by the following projection onto the flow of the graviton *n*-point functions $\partial _t \Gamma _k^{(n)}$. We use the classical Einstein-Hilbert tensor structures $\mathcal {T}^{(n)}(\varvec{p};\Lambda _{n})$ as a basis for our projection operators. Furthermore, we project onto the spin-two transverse-traceless part of the flow, which is numerically dominant. Moreover, it does not depend on the gauge. This transverse-traceless projection operator is then applied to all external graviton legs. The flow of the couplings $\Lambda _{n}$ is then extracted with the help of the momentum-independent part of said tensor structures, namely $\Pi _{\Lambda _n} :=\mathcal {T}^{(n)}(0;\Lambda _{n})/\Lambda _{n}$. For the couplings $G_{n}$ we use $\Pi _{G_n}:=\mathcal {T}^{(n)}(\varvec{p};0)/p^2$. Dividing by $\Lambda _{n}$ and $p^2$ ensures that the projection operators are dimensionless and scale-independent.

In principle, the flow of any *n*-point function depends on all external momenta $p_i, i\in \{1,...,n\}$, where e.g. $p_n$ can be eliminated due to momentum conservation. For the two-point function, the momentum configuration is trivial, and only one momentum squared, $p^2$, needs to be taken into account. In contrast, this dependence becomes increasingly complex for the higher *n*-point functions: The three-point function depends on three parameters (two momenta squared and one angle), the four-point function already depends on six parameters, and so on. To simplify the computations, we use a maximally symmetric $(n-1)$-simplex configuration for all *n*-point-functions, thereby reducing the momentum dependence to a single scalar parameter. This symmetric momentum configuration was already used for the graviton three-point function in [3]. In the context of Yang-Mills theories, this approximation has been shown to be in good agreement with lattice computations on the level of the flow of the propagator [109]. Notably, in the symmetric momentum configuration all external momenta have the same absolute value *p*, and the same angles between each other. The scalar product of any two momenta in this momentum configuration then reads

21 $$\begin{aligned} p_i \cdot p_j = \frac{n \delta _{i j} - 1}{n-1} p^2, \end{aligned}$$

where $\delta _{i j}$ denotes the Kronecker delta. Note that such a symmetric momentum configuration only exists up to the $(d+1)$-point function, where *d* is the dimension of spacetime.

In the following, the expressions $\text {Flow}^{(n)}$ stand for the dimensionless right-hand sides of the flow equations divided by appropriate powers of the wave function renormalisations. More explicitly, we define

22 $$\begin{aligned} \text {Flow}^{(n)}_i(p^2) :=\frac{\partial _t \Gamma ^{(n)}_{i}(p^2) }{Z^{\frac{n}{2}}_{\phi } (p^2) k^{2-n}}, \end{aligned}$$

where the index *i* represents the projection on some tensor structure. In this work, we use the transverse-traceless projection operator $\Pi _\mathrm{TT}$, the projection operators $\Pi _{G_n}$ and $\Pi _{\Lambda _n}$ mentioned earlier for the graviton $n$-point functions, as well as the transverse projection operator $\Pi _\mathrm{T}$ for the ghost propagator. Note that the objects $\text {Flow}^{(n)}_i$ do not contain any explicit factors of the wave function renormalisations $Z_{\phi }$. Instead, their running appears via the anomalous dimensions $\eta _{\phi }$.

**Fig. 2:**
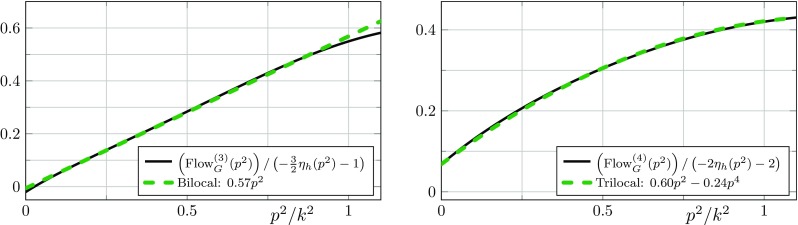
Momentum dependence of the flow of the graviton three-point function (left) and the graviton four-point function (right) divided by $(-\frac{n}{2} \eta _{h}(p^2) - n + 2)$ as defined in (26). The flows are evaluated at $\left( \mu , \lambda _{3}, \lambda _{4}, g_{3}, g_{4} \right) = \left( -0.4, 0.1, -0.1, 0.7, 0.5 \right)$ and $\lambda _{6}=\lambda _{5}=\lambda _{3}$ as well as $g_{6}=g_{5}=g_{4}$. The flows have such a simple polynomial structure as long as all couplings $\lambda _{n}$ remain small, i.e. $|\lambda _{n}|\lesssim 1$. Importantly, the inclusion of a $p^4$ term in the left panel offers no significant improvement. Note that the constant parts of the functions are irrelevant for the beta functions since they are extracted from a different tensor projection. For $p^2>k^2$ the momentum dependence of the flows is not polynomial anymore

Last but not least, we choose to model the regulator functions $R^{\phi _i}$ on the corresponding two-point functions at vanishing mass, i.e.

23 $$\begin{aligned} R^{\phi _i} (p_i^2) = \left. \Gamma ^{(\phi _i \phi _i)} (p_i^2) \right| _{m_{\phi _i} = 0} r_{\phi _i} ( p_i^2/k^2 ). \end{aligned}$$

Here, $r_{\phi _i} ( p_i^2/k^2 )$ denotes the regulator shape function. For all fields in this work, we choose the Litim regulator [113], to wit

24 $$\begin{aligned} r( x ) = \left( x^{-1} - 1 \right) \Theta \left( 1 - x \right) . \end{aligned}$$

This choice allows for analytic flow equations for all couplings that are evaluated at vanishing external momenta.

Furthermore, we introduce the dimensionless couplings

25 $$\begin{aligned} \mu :=M^2 k^{-2}, \quad \lambda _{n} :=\Lambda _{n} k^{-2}, \quad g_{n} :=G_{n} k^2. \end{aligned}$$

At the UV and IR fixed points, the flow of these dimensionless couplings vanishes.

### Momentum dependence of the graviton $n$-point functions

We now investigate the momentum dependence of the flow of the graviton $n$-point functions as defined in (22). We restrict ourselves to the momentum range $0 \le p^2 \le k^2$ as well as to the transverse-traceless part of the graviton $n$-point functions.

The first non-trivial result is that the flows of the graviton three- and four-point functions projected on the tensor structure of the gravitational coupling and divided by $(-\frac{n}{2} \eta _{h}(p^2) - n + 2)$ are well described by a polynomial in $p^2$, provided that the couplings $\lambda _{n}$ are small, i.e.

26 $$\begin{aligned} \frac{\text {Flow}_G^{(3)} (p^2)}{-\frac{3}{2} \eta _{ h}(p^2) - 1}&\approx a_{0} + a_{1}\, p^2 \nonumber \,,\\ \frac{\text {Flow}_G^{(4)} (p^2)}{-2 \eta _{h}(p^2) - 2}&\approx b_{0} + b_{1}\, p^2 + b_{2}\, p^4 \,, \end{aligned}$$

with some constants $a_i$ and $b_i$ that depend on the evaluation point in theory space. This momentum dependence is displayed in Fig. 2. We emphasise that these equations only hold in the momentum range $0 \le p^2 \le k^2$, if the flow is generated by Einstein-Hilbert vertices, and if the constant parts of the vertices are small, i.e. $|\lambda _{n}|\lesssim 1$. If the condition of small $\lambda _{n}$ is violated, then the flow as in (26) is non-polynomial. We did not compute the flow generated by an action including higher curvature terms, however, we suspect that the flow will still be polynomial but possibly of a higher degree.

It is important to note that the graviton three- and four-point functions have a different highest power in $p^2$. This is a second non-trivial result for the following reasons: as already mentioned before, the coupling $g_{3}(p^2)$ has an overlap with $R$ and $R_{\mu \nu }^2$, and higher derivative terms in $f^{(3)}_{R_{\mu \nu }^2}$, but not with any $R^2$ tensor structures in $f^{(3)}_{R^2}$, c.f. (19). For example, the generation of $R_{\mu \nu }^2$ with $P^{(3)}_{R_{\mu \nu }^2}=1$ would manifest itself in a $p^4$-contribution to the flow of the graviton three-point function. Equation (26) and Fig. 2 show that such a $p^4$-contribution as well as higher ones are approximately vanishing. This demonstrates in particular that the generation of $R_{\mu \nu }^2$ is non-trivially suppressed. In other words,

27 $$\begin{aligned} f^{(3)}_{R_{\mu \nu }^2}\approx 0, \end{aligned}$$

where the superscript indicates the three-graviton vertex.

On the other hand, the projection on $g_{4}(p^2)$ overlaps with $R$, $R_{\mu \nu }^2$, $R^2$ tensor structures, and the related higher derivatives terms in $f^{(4)}_{R_{\mu \nu }^2}$ and $f^{(4)}_{R^2}$. It also overlaps with curvature invariants to the third power with covariant tensors such as $f^{(4)}_{R_{\mu \nu }^3}$ and similar ones. Note that it has no overlap with $f^{(4)}_{R^3}$.

Similarly to possible $p^4$-contributions for the three-graviton vertex, $p^6$-contributions and even higher powers in $p^2$ could be generated but are non-trivially suppressed. The $p^4$-contribution to the flow, which is described in (26) and displayed in Fig. 2, could stem from either $R^2$ or $R_{\mu \nu }^2$ tensor structures. Now we use (27). It entails that the graviton three-point vertex does not generate the diffeomorphism invariant term $R_{\mu \nu }^2$ although it has an overlap with it. This excludes $R_{\mu \nu }^2$ as a relevant UV direction, which would otherwise be generated in all vertices. This statement only holds if we exclude non-trivial cancellations of which we have not seen any signature. Accordingly we set

28 $$\begin{aligned} f^{(4)}_{R_{\mu \nu }^2}\approx 0, \end{aligned}$$

and conclude that this $p^4$-contribution or at least its UV-relevant part stems solely from $R^2$. It may be used to determine $f^{(4)}_{R^2}$.

In summary, the above statements about the momentum-dependencies are highly non-trivial and show that $R^2$-contributions are generated while $R_{\mu \nu }^2$ and other higher derivative terms are strongly suppressed. These non-trivial findings also allow us to determine the most efficient way to project precisely onto the couplings of different invariants. This is discussed in Sect. 4.5.

We close this section with a brief discussion of the effect of higher derivative terms on perturbative renormalisability and the potential generation of massive ghost states. As already discussed in [114] in a perturbative setup, it is precisely the $R_{\mu \nu }^2$ term which makes the theory perturbatively renormalisable. However, in this setup it gives rise to negative norm states. On the other hand, the $R^2$ term neither ensures perturbative renormalisability, nor does it generate negative norm states. This is linked to the fact that the $R^2$ term does not contribute to the transverse traceless part of the graviton propagator. Consequently, the non-trivial suppression of $R_{\mu \nu }^2$ tensor structures might be interpreted as a hint that we do not suffer from massive ghost states. However, a fully conclusive investigation requires the access to the pole structure of the graviton propagator, and hence a Wick rotation. Progress in the direction of real-time flows in general theories and gravity has been made e.g. in [25, 50, 115–121].

### Higher order vertices and the background effective action

The results in the last section immediately lead to the question about the importance of the higher-order covariant tensor structures like e.g. $f_{R^n}$ which have no overlap with the graviton *n*-point functions computed in this work. These are potentially relevant for the flows of $G_5$ and $G_6$. These tensors have been dropped in the current work, thus closing our vertex expansion. However, we may utilise previous results obtained within the background field approximation for estimating their importance: first we note that $R^2$ gives rise to a new relevant direction, as we will show in Sect. 5.1. This has also been observed for the background field approximation [10, 14–16, 31]. There it has also been shown that the critical dimensions of the $R^n$-terms approximately follow their canonical counting [31]. Furthermore, our results so far have sustained the qualitative reliability of the background field approximation for all but the most relevant couplings. Indeed, it is the background field-dependence of the regulator that dominates the deviation of the background approximation from the full analysis for the low order vertices, and in particular the mass parameter $\mu$ of the graviton. This field-dependence is less relevant for the higher order terms. Thus, we may qualitatively trust the background field approximation for higher curvature terms. This means that they are of sub-leading importance and can be dropped accordingly.

Finally, the above findings together with those from the literature suggest that an Einstein-Hilbert action is generating a diffeomorphism-invariant $R^2$-term but not an $R_{\mu \nu }^2$ term in the diffeomorphism-invariant background effective action $\Gamma _k[g]=\Gamma _k[g,\phi =0]$. Moreover, no higher derivative terms are generated if a non-trivial wave function renormalisation $Z_h(p^2)$ and graviton mass parameter $\mu =-2\lambda _2$ are taken into account. Note that this only applies for an expansion with $p^2 < k^2$. This is a very interesting finding as it provides strong non-trivial support for the semi-quantitative reliability of the background approximation in terms of an expansion in *R* for spectral values smaller than $k^2$ subject to a resolution of the fluctuating graviton propagator: $\mu$ and $Z_h$ have to be determined from the flows of the fluctuation fields or in terms of the mNIs.

### Flow equations for the couplings

In this section we derive the flow equations for the couplings from the projected *n*-point functions.

The flow equations for $\mu$ and $\eta _{h}(p^2)$ are extracted from the transverse-traceless part of the flow of the graviton two-point function. We evaluate this two-point function at $p^2 = 0$ for $\partial _t \mu$, and bilocally at $- \mu k^2$ and $p^2$ for $\eta _{h}(p^2)$. The algebraic equation for $\eta _{c}(p^2)$ can be obtained directly from the transverse part of the flow of the ghost two-point function. These equations are derived in the same fashion as in [2, 71]. For details see also App. 1.

In the case of the couplings $\lambda _{n}$ and $g_{n}(p^2)$, we project onto the flow of the graviton $n$-point functions. The flow equations for the couplings $\lambda _{n}$ are always obtained at $p^2 = 0$, since $\lambda _{n}$ describes the momentum-independent part of the graviton $n$-point functions.

In the case of the couplings $g_{n}(p^2)$ it is technically challenging to resolve the full momentum dependence in the flow. Thus, we resort to a further approximation of the momentum-dependence. We have checked that this approximation holds quantitatively. First we note that typically FRG-flows are strongly peaked at $q\approx k$ due to the factor $q^3$ from the loop integration and the decay for momenta $q\gtrsim k$ due to $\partial _t R_k(q^2)$. This certainly holds for all the flows considered here. From this we can infer that we extract the leading contribution to the flow diagrams if we feed $g_{n}(k^2)$ back into the diagrams. In consequence we compute only the flow equations for $g_{n}(k^2)$, as they form a closed system of equations within the given approximation.

Conveniently, the momentum dependence of the flow for $g_{3}(p^2)$ is trivial, see Fig. 2 in Sect. 4.3. Hence the approximation discussed above is of quantitative nature, and we obtain precisely the same equation as in [3].

In contrast, the flow of the graviton four-point function exhibits a $p^4$ contribution, implying a non-trivial $g_{4}(p^2)$. Here our approximation is necessary to simplify the computation significantly. The flow equation for $g_{4}(k^2)$ is obtained from a bilocal momentum projection at $p^2=0$ and $p^2=k^2$, and furthermore uses an approximation that relies on the fact that the coupling $\lambda _{4}$ remains small. We refer to this equation as a bilocal equation. It is explicitly displayed in Appendix 1, see (E5). Within our setup this equation gives the best approximation of the vertex flows since it feeds back the most important momentum information into the flow. This further entails that the coupling $g_{4}(k^2)$ includes information about the invariants *R* and $R^2$.

### Disentangling *R* and $R^2$ tensor structures

In this section we present projection operators that disentangle contributions from *R* and ${R^2}$ tensor structures to the flows of the couplings $g_{n}(p^2)$. In the present setup this only allows us to switch off the $R^2$ coupling and thus to check the importance of the $R^2$ coupling.

For the disentanglement, we have to pay attention to two things: First of all, a local momentum projection at $p^2=0$ is very sensitive to small fluctuations and in consequence not very precise with regard to the whole momentum range $0\le p^2 \le k^2$. This was already discussed in [2, 3] and is explicitly shown in Appendix 1. Hence, we have to rely on non-local momentum projections. Here the highest polynomial power of $p^2$, as indicated in (26), dictates the simplest way of projecting on the $p^2$-coefficient. The graviton three-point function is at most quadratic in the external momentum, and consequently it is enough to use a bilocal projection at $p^2 = 0$ and $p^2 = k^2$. The resulting equation is displayed in Appendix 1, see (E6).

The graviton four-point function, on the other hand, has $p^4$ as its highest momentum power, i.e. it is of the form

29 $$\begin{aligned} f(p^2)&= b_0 + b_1\, p^2 + b_2\, p^4, \end{aligned}$$

see also (26). Thus a bilocal momentum projection would not extract the $p^2$ coefficient $b_1$ alone. Instead, we use a trilocal momentum projection at $p^2=0$, $p^2=k^2/2$, and $p^2=k^2$ in order to solve the above equation for $b_1$. I.e., we solve a system of linear equations and obtain

30 $$\begin{aligned} b_1&= - 3 f(0) + 4 f(k^2/2) - f(k^2). \end{aligned}$$

The resulting flow equation is again presented in Appendix 1, see (E7).

For even higher order momentum contributions we would have to use even more points of evaluation. These momentum projections together with the observation of (26) guarantee that we project precisely on the $p^2$ coefficient in the whole momentum range $0\le p^2 \le k^2$.

A natural upgrade of the current approximations amounts to the introduction of a second tensor structure that is orthogonal to the Einstein-Hilbert one in terms of these projections. Within our uniformity assumption this is considered to be sub-leading, and the momentum-dependence of $g_4(p^2)$ takes care of the contribution of the $R^2$ tensor structure $f^{(4)}_{R^2}$. While the orthogonal projection on the respective flow is simple, its back-feeding demands a two tensor structure approximation of the three- and four-graviton vertex in the flow, the implementation of which is deferred to future work.

Here, we only perform a further check of the relevance of the $R^2$ tensor structure. This sustains the fact that the inclusion of the four-graviton vertex with its contribution of the $R^2$ tensor structure leads to an additional UV-relevant direction. To that end we generalise our ansatz for the graviton four-point function such that we can extract a flow equation for both the Einstein-Hilbert tensor structure as well as for the $R^2$ tensor structure. As already mentioned above, we cannot feed the generated coupling back into the flows, since they are given by vertices with Einstein-Hilbert tensor structures. Instead we compute the fixed point value that arises only from the Einstein-Hilbert tensor structures.

As the corresponding ansatz for the transverse-traceless graviton four-point function we choose

31 $$\begin{aligned} \Gamma _k^{(4)}(p^2)&= Z^{2}_{h} (p^2) G_{4} \left( C^{G_{4}}_{\Lambda _{4}} \Lambda _{4} + C^{G_{4}}_{p^2} p^2 + C^{G_{4}}_{\omega _{4}} \Omega _{4} p^4 \right) , \end{aligned}$$

which is precisely the vertex that emerges from the sum of Einstein-Hilbert tensor structure and $R^2$ tensor structure. The related generating diffeomorphism-invariant action for this four-graviton vertex is

32 $$\begin{aligned} S&= S_\text {EH}+ \frac{1}{16 \pi G_N} \int \mathop {\mathrm {d}^{4} x} \sqrt{\det g} \, \Omega \, R^2, \end{aligned}$$

where $S_\text {EH}$ is defined as in (4). The flow of $\Omega _4$ is then obtained by the trilocal momentum projection described below (29). For $b_2$ we obtain

33 $$\begin{aligned} b_2&= 2 f(0) -4 f(k^2/2) +2 f(k^2). \end{aligned}$$

The explicit form of the resulting flow equation for the dimensionless coupling $\omega _{4} :=\Omega _{4} k^2$ is given in Appendix 1, see (E8). Note that in the present approximation, the flows do not depend on the coupling $\omega _{4}$ since it does not feed back into the vertices.

### Computational details

The computations of correlation functions described in this section involve contractions of very large tensor structures. To give a rough estimate: the classical Einstein-Hilbert three-point vertex alone consists of around 200 terms, and the classical graviton propagator of 7 terms. For the box diagram of the flow of the graviton four-point function, displayed in Fig. 1, this results in a total number of approximately $200^4\cdot 7^4 \approx 4\cdot 10^{12}$ terms, if no intermediate simplifications are applied.

These contractions are computed with the help of the symbolic manipulation systems *FORM* [122, 123] and *Mathematica* [124]. For individual tasks, we employ specialised and in part self-developed Mathematica packages. In particular, we use *VertEXpand* [125] and *xPert* [126] for the generation of vertex functions, *DoFun* [127] to obtain symbolic flow equations, and the *FormTracer* [103, 109, 128] to create optimised *FORM* scripts to trace diagrams. Furthermore, we make use of the self-developed framework *TARDIS* [125] facilitating an automated and seamless usage of the aforementioned tools.

## Asymptotic safety

In this section, we discuss the UV fixed point structure of our system. We first present our best result, which includes the tensor structures as presented in Sect. 4.1 and in particular in (18) and (20). The underlying UV-relevant diffeomorphism invariants turn out to be $\Lambda$, *R*, and $R^2$. The $R^2$ coupling is included via the momentum dependence of the gravitational coupling $g_{4}(p^2)$, see Sect. 4.5. As a main result we find an attractive UV fixed point with three attractive directions. The third attractive direction is related to the inclusion of the $R^2$ coupling.

We further analyse the stability of this UV fixed point with respect to the identification of the higher couplings. We also analyse the previous truncation [3] and compare the stability of both truncations. Here we find that the improvement of the truncation increases the stability of the system. In particular, we find a rather large area in the theory space of higher couplings where the UV fixed point exists with three attractive directions throughout.

Lastly, we discuss the importance of the $R^2$ coupling. In Sect. 4.6 we have constructed projection operators that disentangle the contributions from *R* and $R^2$ tensor structures. This allows us to switch off the $R^2$ coupling and compare the stability of the reduced system to that of the full system. We find that the reduced system is significantly less stable, and that the area in the theory space of higher couplings where the fixed point exists is rather small. This highlights the importance of the $R^2$ coupling.

**Fig. 3:**
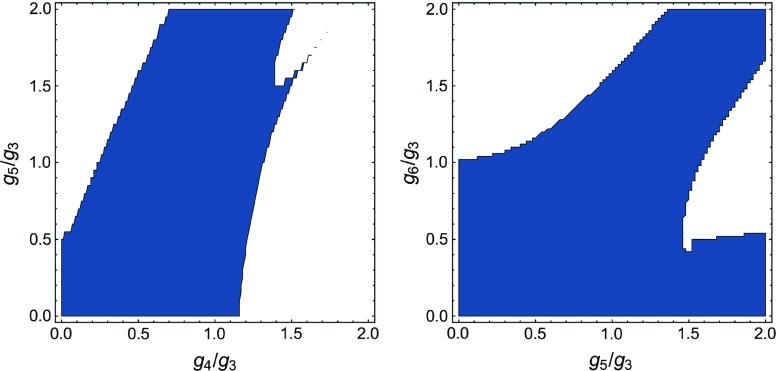
Plots of the existence of an attractive non-trivial UV fixed point (blue) dependent on the higher couplings. Left: The system $(\mu ,\lambda _{3},g_{3})$ [3] dependent on the higher couplings $g_{4}$ and $g_{5}$. Right: The system $(\mu ,\lambda _{3},\lambda _{4},g_{3},g_{4})$ dependent on the higher couplings $g_{5}$ and $g_{6}$. The higher couplings $\lambda _{n>n_{\mathrm {max}}}$ are always identified with $\lambda _{3}$. The blue area marks the region where an attractive UV fixed point was found. At the border of this area the fixed point either vanishes into the complex plane or loses its attractiveness. In both systems the area where the fixed point exists is rather large and contains the identification $g_{n>n_{\mathrm {max}}}=g_{3}$. Conveniently, the area increases for the better truncation, indicating that the system becomes more stable with an improvement of the truncation. The number of attractive directions is uniformly two in the left panel and three in the right panel

### UV fixed point

In this section we display the UV fixed point structure of our full system. This means that we feed back the generated $R^2$ coupling via the momentum dependence of the gravitational coupling $g_{4}(p^2)$, as discussed in Sect. 4.5. Fixed points are by definition points where the flows of the dimensionless couplings vanish. In consequence, we look for the roots of the Eqs. (E1), (E4), (E6), and (E5). We use the identification scheme $g_{6}=g_{5}=g_{4}$ and $\lambda _{6}=\lambda _{5}=\lambda _{3}$. We find a UV fixed point at the values

34 $$\begin{aligned} \left( \mu ^*, \lambda _{3}^*, \lambda _{4}^*, g_{3}^*, g_{4}^* \right)&= \left( -0.45,\, 0.12, 0.028,\, 0.83,\, 0.57 \right) \,. \end{aligned}$$

The fixed point values are similar to those of the previous truncation [3]. The biggest change concerns the graviton mass parameter, which is now less negative and thus further away from its pole. Moreover, it is remarkable that the new couplings $\lambda _{4}$ and $g_{4}$ are close to their lower counterparts $\lambda _{3}$ and $g_{3}$, but not at precisely the same values. Since we use the difference between these couplings to parameterise the breaking of diffeomorphism invariance, this is more or less what we expected. This issue is further discussed in the next section.

We do not have access to the full stability matrix of the UV fixed point due to the unknown flow equations of the higher couplings. For this reason, we discuss two different approximations of the stability matrix. The main difference between these two approximations concerns the order of taking the derivatives and identifying the higher couplings, which is explained in more detail in Appendix 1. We argue that in a well converged approximation scheme the most relevant critical exponents should not depend on the approximation of the stability matrix. Thus, we can use the two different approximations to judge the quality of the current level of truncation. In this work, we define the critical exponents as the eigenvalues of the stability matrix without a minus sign. We call the critical exponents of the first approximation $\bar{\theta }_i$, and the ones of the second approximation $\tilde{\theta }_i$. The critical exponents using the first approximation are given by

35 $$\begin{aligned} \bar{\theta }_i&= (-4.7,\; -2.0 \pm 3.1 \mathrm {i},\; 2.9,\; 8.0 ), \end{aligned}$$

while the critical exponents using the second approximation are

36 $$\begin{aligned} {\tilde{\theta }}_i&= (-5.0,\; -0.37 \pm 2.4 \mathrm {i},\; 5.6,\; 7.9). \end{aligned}$$

Hence this fixed point has three attractive directions in both approximations of the stability matrix. The third attractive direction compared to the system of the graviton three-point function [3] is related to the fact that the graviton four-point function has an overlap with $R^2$, which we feed back via the momentum dependence of the gravitational coupling $g_{4}(p^2)$. The $R^2$ coupling has also been relevant in earlier computations with the background field approximation [10, 14–16, 31]. In addition, note that the most attractive eigenvalue is almost identical in both approximations of the stability matrix. This is a positive sign towards convergence since it is expected that the lowest eigenvalue is the first that converges, c.f. Appendix 1.

Furthermore, the anomalous dimensions at the UV fixed point read

37 $$\begin{aligned} (\eta _{h}^*(0), \eta _{h}^*(k^2))&= (0.56 ,\; 0.079 )\,, \nonumber \\ (\eta _{c}^*(0), \eta _{c}^*(k^2))&= ( -1.28 ,\; -1.53 )\,, \end{aligned}$$

where we have chosen to display the anomalous dimensions at the momenta that feed back into the flow. All anomalous dimensions stay well below the reliability bound $\eta _{\phi _i}(p^2)<2$, as introduced in [71].

### Stability

In the following we investigate the UV fixed point from the previous section by varying the identification of the higher couplings. Again we look for the roots of the Eqs. (E1), (E4), (E6), and (E5). These equations however still depend on the higher couplings $g_{5}$, $g_{6}$, $\lambda _{5}$, and $\lambda _{6}$. We have to identify these couplings with the lower ones or set them to constants in order to close the flow equations.

It is a natural choice to simply set these higher couplings equal to lower ones, e.g. $g_{6}=g_{5}=g_{3}$ and $\lambda _{6}=\lambda _{5}=\lambda _{3}$, as done in the previous section. The couplings would fulfil this relation exactly in a fully diffeomorphism invariant setup. However, such a diffeomorphism invariant setup is not at hand. In fact, we can parameterise the breaking of diffeomorphism invariance via these couplings, e.g. by writing $g_{n} = g_{3} + \Delta _{g_n}$. Here we have designated $g_{3}$ as a reference coupling since it is the lowest genuine gravitational coupling. For this reason, it is also the most converged gravitational coupling within this vertex expansion, thus justifying this choice. In general we expect $\Delta _{g_n}$ to be small and in consequence we vary the identification of the higher couplings only in this part of the theory space of higher couplings. The quantity $\Delta _{g_4}$ is indeed small at the UV fixed point presented in the last section, see (34). More precisely, it takes the value $|\Delta _{g_4}/g_{3}| \approx 0.3$ at this UV fixed point.

In this analysis we choose to identify

38 $$\begin{aligned} g_{5}&= \alpha _1\, g_{3},&g_{6}&= \alpha _2\, g_{3}, \end{aligned}$$

and $\lambda _{6}=\lambda _{5}=\lambda _{3}$ for simplicity, and investigate the existence of the UV fixed point as a function of the parameters $\alpha _1$ and $\alpha _2$. In Fig. 3 the area where an attractive UV fixed point exists is displayed in blue. In the left panel, this is done for the previous truncation $(\mu ,\lambda _{3},g_{3})$ [3], and in the right panel for the current truncation $(\mu ,\lambda _{3},\lambda _{4},g_{3},g_{4})$. At the border of the blue area the UV fixed point either vanishes into the complex plane or loses its attractiveness. Remarkably, both areas are rather large, suggesting that the existence of the UV fixed point is quite stable. Even more conveniently, the area increases with the improved truncation, suggesting that the system is heading towards a converging limit. Note that the number of attractive directions of the UV fixed point is constant throughout the blue areas, namely two in the left panel and three in the right panel.

We further analyse the fixed point values that occur within the blue area in the right panel of Fig. 3. Interestingly, the fixed point values are rather stable throughout the whole area where the UV fixed point exists. More precisely, they stay within the following intervals:

$$\begin{aligned} \mu ^*&\in [-0.72,\,-0.19] \, \\ \lambda _{3}^*&\in [-0.018,\,0.29] \,\\ \lambda _{4}^*&\in [-1.2,\,0.12] \,\\ g_{3}^*&\in [0.22,\,1.4] \, \\ g_{4}^*&\in [0.11,\,0.97]. \end{aligned}$$

Hence, in particular the fixed point value of $\lambda _{3}$ is already confined to a very small interval, and also a very small number. The latter is important since some of our approximations rely on the fact that the $\lambda _{n}$ are small, see Sect. 4.5. The fact that $\lambda _{4}^*$ is varying more strongly than $\lambda _{3}^*$ is not surprising since we expect $\lambda _{3}$ to be better converged, being a lower coupling. The fixed point values of $g_{3}$ and $g_{4}$ seem to try to compensate the change induced by the identification. Thus, $g_{3}^*$ and $g_{4}^*$ become larger towards the identification $g_{6}=g_{5}=0$ and smaller towards $g_{6}=g_{5}=2g_{3}$. The shape of the area in the left panel in particular suggests the relation $g_{4}^*<g_{3}^*$, which is fulfilled by the improved truncation almost throughout the whole area where the fixed point exists. This is indeed a non-trivial prediction that has been fulfilled by our approximation scheme.

A further study of the dependence of the UV fixed point properties on the choice of identification is given in Appendix 1.

**Fig. 4:**
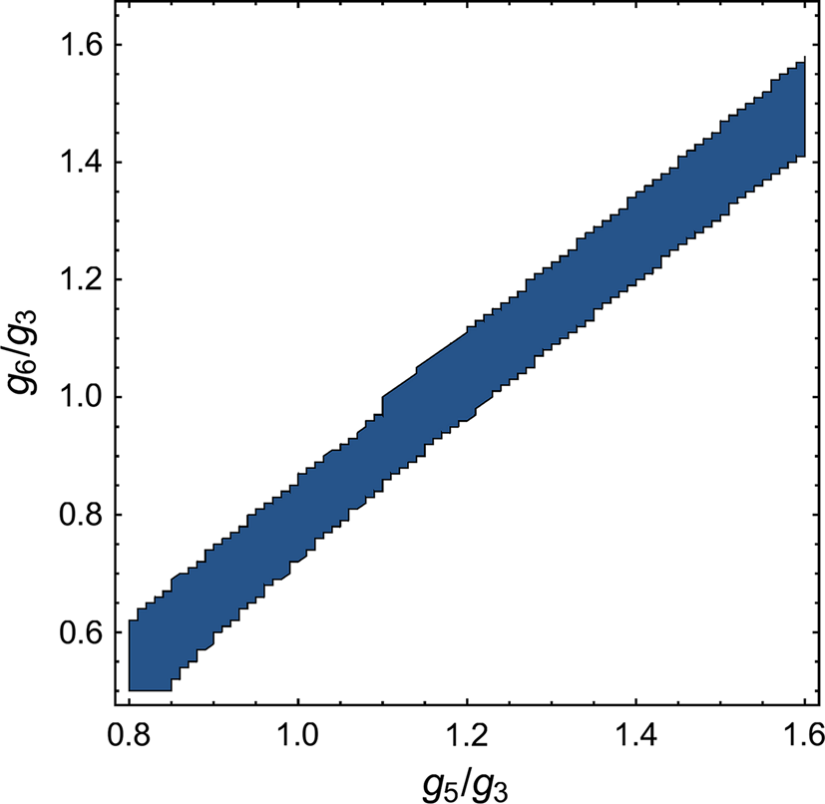
Plot of the existence of an attractive non-trivial UV fixed point (blue) dependent on the higher couplings $g_{5}$ and $g_{6}$. Here, the trilocal equation for the gravitational coupling $g_{4}$ was used, which allows us to switch off the $R^2$ coupling. We found two different fixed points with rather similar fixed point values. Each fixed point has its own area of existence in the theory space of the higher couplings. The blue area marks the unified area of both fixed points. Nevertheless, the area is significantly smaller than the areas displayed in Fig. 3. This reflects the importance of the $R^2$ coupling

### Importance of the $R^2$ tensor structure

In the previous subsection we have fed back the $R^2$ contributions to the flow via the momentum-dependent gravitational coupling $g_{4}(p^2)$. In order to check the quality of our approximation and to investigate the influence of the $R^2$ tensor structure on the fixed point structure of the system, we switch off the $R^2$ contribution in this section. We do the latter by projecting onto the $p^2$ part of the flow via a trilocal momentum projection scheme, cf. Sects. 4.3 and  4.6. This is both an examination of the influence of $R^2$ on the results presented in the previous subsections, as well as a proof of concept for disentangling the tensor structures of different invariants. Our analysis in this subsection suggests that leaving out the contribution of $R^2$ leads to significantly less stable results.

**Fig. 5:**
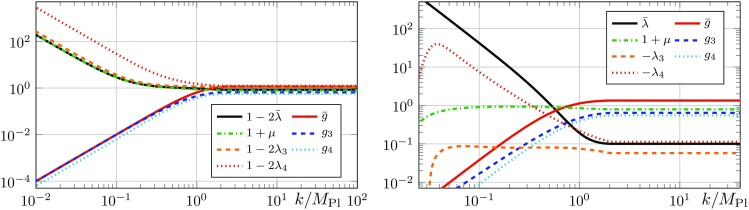
Examples of UV finite trajectories from the UV fixed point (34) towards the IR. In the left panel all couplings scale classically below the Planck scale and reach their UV fixed point values shortly above the Planck scale. In the right panel some couplings show non-classical behaviour even below the Planck scale, which is triggered by the graviton mass parameter $\mu$ flowing towards the pole of the graviton propagator at $\mu = -1$. However, in this case the numerics break down at $k \approx 0.02 M_{\text {Pl}}$ due to competing orders of the factor $(1 + \mu )$ close to the singularity at $\mu = -1$. The trajectories in both panels correspond to theories that behave like classical general relativity in the IR. Note that some couplings are plotted shifted or with a minus sign in order to keep them positive over the whole range

In Fig. 4 we display the result for the same analysis as in the previous section, but with the trilocal equation (E7) for $g_{4}$ instead. We find two fixed points with rather similar fixed point values. However, we are only interested in identifying the area in the theory space of the higher couplings where at least one UV fixed point exits. Thus, we unify both areas and obtain the blue area displayed in Fig. 4. This area forms a rather narrow band whose total area is significantly smaller than for the momentum dependent gravitational coupling $g_{4}(p^2)$, c.f. Fig. 3. The identification $g_{6}=g_{5}=g_{3}$ also does not lie within these regions, but just outside of them. Since we switched off the $R^2$ contribution, a less stable fixed point structure was to be expected, and consequently these results highlight the importance of the $R^2$ coupling.

**Table 1:** Properties of the non-trivial UV fixed point for different orders of the vertex expansion scheme, computed for momentum dependent anomalous dimensions $\eta _{\phi _i}(p^2)$ and bilocally projected Newton’s couplings $g_{n}(k^2)$. The critical exponents $\bar{\theta }_i$ and ${\tilde{\theta }}_i$ stem from two different approximation of the stability matrix as discussed in Appendix 1. The fixed points are computed with the identifications $g_{6}=g_{5}=g_{\text {max}}$ and $\lambda _{6}=\lambda _{5}=\lambda _{3}$. We observe that the fixed point values are only varying mildly between the different orders of the vertex expansion. Notably, if we compare the critical exponents of the two approximations of the stability matrix, we observe that the difference becomes smaller with an increasing order of the vertex expansion. This is precisely what one would expect of a systematic approximation scheme that is approaching a converging limit

System	$\mu ^*$	$\lambda _{3}^*$	$\lambda _{4}^*$	$g_{3}^*$	$g_{4}^*$	${\bar{\theta }}_i$					${\tilde{\theta }}_i$				
$\mu$, $g_{3}$, $\lambda _{3}$	$-\,0.57$	0.095		0.62		$-\,1.3 \pm 4.1\mathrm {i}$		12			$-\,7.3$	3.5	7.4		
$\mu$, $g_{3}$, $\lambda _{3}$, $g_{4}$	$-\,0.53$	0.086		0.74	0.67	$-\,2.1 \pm 3.8 \mathrm {i}$		3.6	11		$-\,0.75 \pm 1.5 \mathrm {i}$		$7.8 \pm 3.5\mathrm {i}$		
$\mu$, $g_{3}$, $\lambda _{3}$, $\lambda _{4}$	$-\,0.58$	0.17	0.032	0.48		$-\,4.1$	$-\,0.35 \pm 2.6 \mathrm {i}$		8.3		$-\,6.2$	$-\,1.8$	3.4	8.8	
$\mu$, $g_{3}$, $\lambda _{3}$, $g_{4}$, $\lambda _{4}$	$-\,0.45$	0.12	0.028	0.83	0.57	$-\,4.7$	$-\,2.0 \pm 3.1 \mathrm {i}$		2.9	8.0	$-\,5.0$	$-\,0.37 \pm 2.4 \mathrm {i}$		5.6	7.9

## IR behaviour

In this section, we discuss the IR behaviour of the present theory of quantum gravity. We only consider trajectories that lie within the UV critical hypersurface, i.e. trajectories that are UV finite, and which end at the UV fixed point presented in (34) for $k\rightarrow \infty$. In this section we use the analytic flow equations given in Appendix 1 for simplicity, and set the anomalous dimensions to zero, i.e. $\eta _{\phi }=0$. This approximation gives qualitatively similar results, as discussed in Appendix 1.

In the IR, it is particularly interesting to examine the background couplings $\bar{g}$ and $\bar{\lambda }$. In the limit $k\rightarrow 0$ the regulator vanishes by construction and the diffeomorphism invariance of the background couplings is restored. Hence they become observables of the theory. The flow equations for the background couplings are displayed in Appendix 1.

In general we look for trajectories that correspond to classical general relativity in the IR. This implies that the quantum contributions to the background couplings vanish and in consequence that they scale classically according to their mass dimension. The classical scaling is described by

39 $$\begin{aligned} \bar{g},\, g_{3},\, g_{4}\, \sim k^2, \quad \quad \bar{\lambda },\, \mu ,\, \lambda _{3},\, \lambda _{4}\, \sim k^{-2} \,. \end{aligned}$$

We use the classical scaling in the flow from the UV fixed point to the IR in order to set the scale *k* in units of the Planck mass $M_{\text {Pl}}$. We need to find a large enough regime where $\bar{g}\sim k^{-2}$. This entails that Newton’s coupling is a constant in this regime and sets the scale *k* via $G_N= M_{\text {Pl}}^{-2} = \bar{g}k^{-2}$.

In Fig. 5, two exemplary trajectories are displayed. In the left panel all couplings scale classically below the Planck scale and reach their UV fixed point values shortly above the Planck scale. All quantum contributions are suppressed simply by the fact that $\mu \rightarrow \infty$. In the right panel on the other hand some couplings exhibit a non-classical behaviour even below the Planck scale, which is triggered by the graviton mass parameter $\mu$ flowing towards the pole of the graviton propagator at $\mu = -1$. This entails that the dimensionful graviton mass parameter $M^2=\mu k^2$ is vanishing in the IR. This IR behaviour is analogous to the one observed in [2], and recently also [50]. Remarkably, not only $\mu$ is behaving non-classically but also $\lambda _{3}$, even though it is not restricted by any pole. However, in this scenario the numerics break down at $k \approx 0.02 M_{\text {Pl}}$ due to competing orders of the factor $(1 + \mu )$ close to the singularity at $\mu = -1$.

In the left panel we have tuned the background couplings $\bar{g}$ and $\bar{\lambda }$ so that they are equal to the lowest corresponding fluctuation coupling in the IR, i.e. $\bar{g}=g_{3}$ and $\bar{\lambda }=\lambda _{2}=-\mu /2$ for $k\ll M_{\text {Pl}}$. This is equivalent to solving a trivial version of the Nielsen identities (NIs). Since all quantum contributions are suppressed by the graviton mass parameter going to infinity in the IR, $\mu \rightarrow \infty$, the NI in (2) reduces to

$$ \begin{aligned} \frac{\delta \Gamma [{\bar{g}},h]}{\delta {\bar{g}}} =\frac{\delta \Gamma [{\bar{g}}, h]}{\delta h} \quad \quad \text {for} \quad \mu \rightarrow \infty \quad \& \quad k\rightarrow 0 \,. \end{aligned}$$

In consequence, we should see that all couplings coincide in this limit, $\bar{g}=g_{n}$ and $\bar{\lambda }=\lambda _{n}$. This is not the case in the left panel of Fig. 5 since we have only fine-tuned the background couplings, and thus we have two further degrees of freedom that could be used for fine-tuning, stemming from the three dimensional UV critical hypersurface. We postpone this fine-tuning problem to future work.

In summary, we find different types of trajectories that correspond to classical general relativity in the IR. The main difference lies in the behaviour of the graviton mass parameter $\mu$, which flows to infinity in one case and to minus one in the other case. Both scenarios are equivalent to general relativity in the end, in particular since only the background couplings become observables in the limit $k\rightarrow 0$.

## Towards apparent convergence

In this section we discuss and summarise the findings of this work concerning apparent convergence. On the one hand, the order of our vertex expansion is not yet high enough to fully judge whether the system approaches a converging limit. Nevertheless, we have collected several promising first hints that we want to present in the following.

In this work we have introduced two different approximations to the stability matrix, as presented in Appendix 1. We have argued that in a well converged approximation scheme the most relevant critical exponents should not depend on the approximation of the stability matrix. In Table 1 we display the UV fixed point properties for different orders of the vertex expansion. The first system is without the graviton four-point function and exactly the same as in [3]. Then we look at systems where we add either only an equation for $g_{4}(k^2)$ (c.f. (E5)), or only an equation for $\lambda _{4}$ (c.f. (E4)). Lastly, we display our best truncation including all couplings up to the graviton four-point function, see Sect. 5.1. We observe that the fixed point values of the couplings vary only mildly with an improving truncation, although there is no clear pattern to those variations. The most important piece of information is the difference between the critical exponents from the two different approximations of the stability matrix. While the difference is rather large in the truncation of the graviton three-point function, it is becoming smaller with each improvement of the truncation. At the level of the graviton four-point function, the critical exponents show only a small difference. This is precisely what we expect, and thus we interpret this as a sign that the system is approaching a converging limit.

Another important piece of information comes from the stability of the UV fixed point under different closures of the flow equation. In a well converged expansion scheme, the properties of the UV fixed point should be completely insensitive to the details of the closure of the flow equation. We have performed this analysis in Sect. 5.2. We observed that the area in which the UV fixed point exists in the theory space of higher couplings is indeed increasing with the improvement of the truncation. Furthermore, we saw that the UV fixed point values are confined to small intervals. We again interpret this as a sign that the system is approaching a converging limit.

In summary, we have already seen several signatures of apparent convergence although we are only at the level of the graviton four-point function within the present systematic expansion scheme. This suggests that we are on a promising path and that the present setup will eventually lead to a converging limit.

## Summary

We have investigated quantum gravity with a vertex expansion and included propagator and vertex flows up to the graviton four-point function. The setup properly disentangles background and fluctuation fields and, for the first time, allows to compare two genuine Newton’s couplings stemming from different vertex flows. Moreover, with the current truncation we have closed the flow of the graviton propagator: all vertices and propagators involved are computed from their own flows.

As a first non-trivial result we have observed that the vertex flows of the graviton three-point and four-point functions, in the sense of (26), are well described by a polynomial in $p^2$ within the whole momentum range $0\le p^2 \le k^2$. The projection used for the flows takes into account the $R$, $R^2$ and $R_{\mu \nu }^2$ tensor structures as well as higher order invariants with covariant momentum dependencies. Importantly, it is orthogonal to the $R^2$ tensor structure for the graviton three-point function, but includes it for the graviton four-point function. We have shown that the highest momentum power contributing to the graviton three-point function is $p^2$. Therefore, $R_{\mu \nu }^2$ and higher derivative terms do not contribute to the graviton three-point function. Thus, in particular $R_{\mu \nu }^2$ is excluded as a UV-relevant direction. On the other hand, the flow of the graviton four-point function shows $p^4$ as its highest momentum power. Together with the three-point function result we infer that $R^2$ is UV-relevant and contributes to the graviton four-point function. This is a very interesting and highly non-trivial result.

At the moment, we cannot make final statements about higher $R^n$ terms directly from our analysis. Nonetheless, predictions can be made with a combination of the results presented here and previous ones obtained within the background field approximation as well as the vertex expansion: Firstly, our work sustains the qualitative reliability of background field or mixed approximations for all but the most relevant couplings. We have seen that the range of allowed Newton’s couplings stemming from *n*-graviton vertices is growing with the level of the approximation. Moreover, in [71, 74] it has been shown that already the substitution of the most relevant operator, the mass parameter $\mu$, in a mixed computation with that in the full vertex expansion stabilises the results in a particular matter gravity system. Hence, this gives us some trust in the qualitative results for higher $R^n$ terms in the background field approximation. In [31] the $f(R)$- potential has been computed polynomially up to $R^{34}$, and the relevance of these operators follows the perturbative counting closely. Accordingly it is quite probable that the higher $R^n$ will turn out to be irrelevant in the full vertex expansion as well.

Based on the above observations we have also constructed projection operators that properly disentangle the contributions of different diffeomorphism-invariant tensor structures. This allowed us to switch off the $R^2$ coupling in order to analyse its importance for the system. In this case, we are led to an unstable system, which highlights the importance of the $R^2$ coupling for the asymptotic safety scenario. In the present work we include the $R^2$ contributions via the momentum dependence of the gravitational coupling $g_{4}(p^2)$, leading to a very stable system in the UV.

In the full system with $R^2$ contributions we found an attractive UV fixed point with three attractive directions and two repulsive directions. The third attractive direction can be explained due to the overlap with $R^2$, and is in agreement with previous $R^2$ studies in the background field approximation [10, 14–16, 31]. We investigated the stability of this UV fixed point with respect to changes of the identification of the higher couplings and compared it to the stability of the previous truncation without the graviton four-point function. We characterised the stability via the area of existence in the theory space of higher couplings, and remarkably this area increased with the improved truncation. We interpret this as a sign that the systematic approximation scheme is approaching a converging limit.

Furthermore, we investigated the IR behaviour and found trajectories that connect the UV fixed point with classical general relativity. In particular, we found two different types of such trajectories. In the first category all couplings, including background and fluctuation couplings, scale classically according to their mass dimension below the Planck scale. In consequence the Nielsen identities become trivial in this regime and we can solve them in the IR. In the second category, the graviton mass parameter and the coupling $\lambda _{3}$ scale non-classically below the Planck scale, which is triggered by the graviton mass parameter flowing towards the pole of the graviton propagator $\mu \rightarrow -1$. In summary, the IR behaviour was found to be very similar to [2], and recently also [50].

Lastly, we discussed signs of apparent convergence in the present system by comparing the results to previous truncations. As mentioned before, we observed that the present system is more stable and less sensitive to the closure of the flow equation, which is expected from a converging system. We furthermore used two different approximations of the stability matrix and argued that the critical exponents belonging to the most attractive directions should not differ in a well converged expansion. Indeed we found that the difference of the critical exponents is decreasing with an improvement of the truncation. We interpret this as a sign towards convergence.

In the present approximation we have taken the $\Lambda$, $R$ and $R^2$ tensor structures into account. Furthermore, we have shown that the higher derivative tensor structures and the $R_{\mu \nu }^2$ tensor structure are suppressed. There are also very strong indications for the irrelevance of the higher orders in $R^n$. Altogether this suggests that the natural extension of the present work towards apparent convergence consists primarily of the inclusion of all tensor structures of the vertices and propagators on the given level $n=4$ of the vertex expansion. In particular, this concerns the inclusion of the $R^2$ tensor structure with the orthogonal projection devised in the present work. Moreover, the different graviton modes should be furnished with their separate dispersion or wave function renormalisation. This is well in reach with the current technical status of the programming code and its implementation. Then, selected tensor structures of higher vertices could be used for further tests of apparent convergence. We hope to report on this in the near future.

## Figures and Tables

**Fig. 6:**
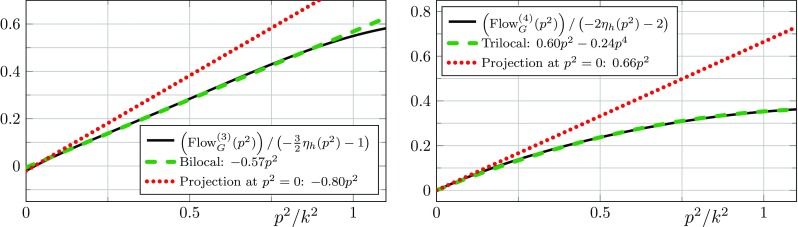
Fit of a local momentum projection at $p^2=0$ to the momentum dependence of the flow of the graviton three-point function (left) and the graviton four-point function (right) divided by $(-\frac{n}{2} \eta _{h}(p^2) - n + 2)$ as defined in (26). The flows are evaluated at $\left( \mu , \lambda _{3}, \lambda _{4}, g_{3}, g_{4} \right) = \left( -0.4, 0.1, -0.1, 0.7, 0.5 \right)$ and $\lambda _{6}=\lambda _{5}=\lambda _{3}$ as well as $g_{6}=g_{5}=g_{4}$, i.e. the same values as in Fig. 2 where a non-local momentum projection was used. In comparison to the non-local momentum projection, the local momentum projection does *not* capture the correct momentum dependence in the whole momentum range $0\le p^2 \le k^2$ since it is sensitive to local momentum fluctuations. Furthermore, it is technically very challenging to project on the $p^4$-term due to IR singularities. Note again that the constant parts of the flows are irrelevant for the beta functions since they are extracted from a different tensor projection

## Appendix A: Approximations of the stability matrix

The stability matrix $B$ is defined as the Jacobi matrix of the flow equations for all couplings $\alpha _{i}$. Mathematically, it is given by

A1 $$\begin{aligned} B_{i j} :=\partial _{\alpha _{j}} \partial _t \alpha _{i}. \end{aligned}$$

In this work, the critical exponents of a fixed point are defined as the eigenvalues of the stability matrix evaluated at this fixed point. In our setup, the stability matrix is infinite dimensional since it is spanned by all couplings $\lambda _{n}$ and $g_{n}(p^2)$. Note that one momentum dependent coupling alone would already be enough to render the stability matrix infinite dimensional.

In the present work, only couplings up to order six appear in the flow. We furthermore do not resolve the full momentum dependence of the couplings. Thus, we have already rendered the stability matrix finite. Nevertheless, the full stability matrix is not known since the flows of the fifth- and sixth order couplings are unknown and would depend on further higher couplings anyway. In consequence, we have to make an approximation of the stability matrix in order to obtain the critical exponents. Note that we also have to make an approximation of the flow itself in order to close it. Naturally, these approximations are related.

In the following we present two different approximations of the stability matrix. We further argue that these approximations should give approximately the same values for the most relevant critical exponents if the expansion scheme is already well converged.

The approximation of the flow is related to its closure and describes how the higher couplings are identified with the lower ones. In the following, we call this process identification scheme and denote it by $|_{\text {id.}}$. The two different approximations of the stability matrix are distinguished by the sequence of taking the derivatives and applying this identification scheme. In the first approximation, the identification is performed *before* taking the derivatives:

A2 $$\begin{aligned} {{\bar{B}}}_{i j} :=\partial _{\alpha _{j}} (\partial _t \alpha _{i}|_{\text {id.}}). \end{aligned}$$

The critical exponents that correspond to this approximation represent the critical exponents that belong to the computed phase diagram of the theory.

In the second approximation, the identification is performed *after* taking the derivatives, to wit

A3 $$\begin{aligned} \widetilde{B}_{i j} :=(\partial _{\alpha _{j}} \partial _t \alpha _{i}) |_{\text {id.}}\,. \end{aligned}$$

This approximation is more closely related to the full stability matrix in the sense that it respects the fact that the higher couplings in the full system do not coincide with the lower ones. Note that these two different approximations only differ if we choose a non-trivial identification scheme, i.e. if the higher couplings are functions of the lower ones.

If the expansion scheme is well converged, then the contributions of the higher couplings to the flow of the lower couplings are small, e.g. $(\partial _{\lambda _{n_{\text {max}}+2}} \lambda _{n_{\text {max}}})|_{\text {FP}}\approx 0$. In this case, the most relevant eigenvalues of the stability matrices $\bar{B}$ and $\widetilde{B}$ coincide approximately. The stabilisation of the most relevant eigenvalues was also observed in an expansion in $R^n$ in [31]. In consequence, a huge deviation in the most relevant eigenvalues of both approximations would clearly indicate a lack of convergence. For this reason, we use the comparison of the different approximations as a first check of convergence.

**Table 2:** Properties of the non-trivial UV fixed point for different identification schemes, i.e. different closures of the flow equations, as discussed in Appendix 1. The flow equations are computed with momentum dependent anomalous dimensions $\eta _{\phi _i}$ and bilocally projected Newton’s couplings $g_{n}(k^2)$. The critical exponents $\bar{\theta }_i$ and ${\tilde{\theta }}_i$ stem from two different approximation of the stability matrix as discussed in Appendix 1. An attractive UV fixed point is found in most identification schemes with mildly varying fixed point values. In the first approximation of the stability matrix we always find three attractive directions, while in the second approximation of the stability matrix we find one or three attractive directions, since the real part of one complex pair of eigenvalues is quite close to zero. These results suggest that the present system is rather stable under change of the closure of the flow equations. In the case of the single identification without a physical UV fixed point we found that it had in fact just vanished in the complex plane

Identification scheme	$\mu ^*$	$\lambda _{3}^*$	$\lambda _{4}^*$	$g_{3}^*$	$g_{4}^*$	${\bar{\theta }}_i$					${\tilde{\theta }}_i$				
$g_{n>4}\rightarrow g_{3}$, $\lambda _{n>4}\rightarrow \lambda _{3}$	$-\,0.48$	0.092	0.0077	0.62	0.53	$-\,5.0$	$-\,1.3 \pm 3.4\mathrm {i}$		3.7	10	$-\,4.2$	$0.62 \pm 1.8\mathrm {i}$		4.7	9.3
$g_{n>4}\rightarrow g_{4}$, $\lambda _{n>4}\rightarrow \lambda _{3}$	$-\,0.45$	0.12	0.028	0.83	0.57	$-\,4.7$	$-\,2.0 \pm 3.1 \mathrm {i}$		2.9	8.0	$-\,5.0$	$-\,0.37 \pm 2.4 \mathrm {i}$		5.6	7.9
$g_{n>4}\rightarrow g_{4}$, $\lambda _{n>4}\rightarrow \lambda _{4}$	Physical UV fixed point not found														
$g_{n>4}\rightarrow g_{4}$, $\lambda _{6}\rightarrow \lambda _{4}$, $\lambda _{5}\rightarrow \lambda _{3}$	$-\,0.49$	0.086	0.027	0.64	0.56	$-\,8.7$	$-\,1.4 \pm 3.7 \mathrm {i}$		4.3	11	$-\,5.0$	$0.46 \pm 2.0 \mathrm {i}$		5.5	11

## Appendix B: Background couplings

In this section we present the flow equations for the background couplings $\bar{g}$ and $\bar{\lambda }$. They are in particular interesting in the limit $k \rightarrow 0$ where they become observables. In this limit, the regulator term vanishes by construction and diffeomorphism invariance is restored, which implies that these couplings can be interpreted as physical observables only for vanishing *k*.

For notational convenience we reintroduce the coupling $\lambda _2 = - \mu /2$. Following [44], we compute the flow of the background couplings with a curvature expansion on an Einstein space. We use a York-decomposition [129, 130] and field redefinitions [9, 57] to cancel the non-trivial Jacobians. The resulting flow equations are given by

B1 $$\begin{aligned} \partial _t {{\bar{g}}}&= 2 {\bar{g}} - {\bar{g}}^2 f_{R^1}(\lambda _2;\eta _\phi )\,, \nonumber \\ \partial _t{\bar{\lambda }}&= -4 {\bar{\lambda }} + {\bar{\lambda }} \frac{\partial _t{{\bar{g}}}}{{\bar{g}}}+ {\bar{g}} f_{R^0}(\lambda _2;\eta _\phi ), \end{aligned}$$

where the functions $f_{R^0}$ and $f_{R^1}$ read

B2 $$\begin{aligned}&f_{R^0}(\lambda _2;\eta _\phi ) = \frac{1}{24 \pi } \nonumber \\&\quad \times \left( \frac{(10-8 \lambda _2)(6-\eta _h(k^2))}{1 - 2 \lambda _2}-8 (6-\eta _c(k^2))\right) \,,\nonumber \\&f_{R^1}(\lambda _2;\eta _\phi ) = \frac{1}{24 \pi }\nonumber \\&\quad \times \left( \frac{93+204 \lambda _2-300 \lambda _2^2-\eta _h(k^2) \left( 17+36 \lambda _2-60 \lambda _2^2\right) }{3 (1 -2 \lambda _2)^2} \right. \nonumber \\&\quad \left. + 10 (5-\eta _c(k^2))\right) . \end{aligned}$$

In consequence the fixed point equations for the background couplings are given by

B3 $$\begin{aligned} \bar{g}^*&= \frac{2}{f_{R^1}(\lambda _{2}^*,\eta _\phi ^*)}\,, \nonumber \\ \bar{\lambda }^*&= \frac{f_{R^0}(\lambda _{2}^*,\eta _\phi ^*)}{2 f_{R^1}(\lambda _{2}^*,\eta _\phi ^*)}. \end{aligned}$$

Note that the background couplings are non-dynamical, i.e. they do not influence any other coupling. Furthermore, the background couplings only depend on the couplings of the two-point function. Hence only the graviton mass parameter $\mu$ (or equivalently $\lambda _2$) and the anomalous dimensions $\eta _h$ and $\eta _c$ directly affect them.

## Appendix C: Dependence on the identification scheme

The flow of each *n*-point function depends on the couplings of the $(n+1)$-point function and the $(n+2)$-point function, see Fig. 1. For the highest couplings, we consequently do not have a flow equation at hand. In our setup, these are the couplings of the five- and six-point function, i.e. $\lambda _{5}$, $\lambda _{6}$, $g_{5}$, and $g_{6}$. In order to close the flow of our system, we need to make an ansatz for these higher order couplings. A natural choice is one that is close to diffeomorphism invariance, i.e. to identify these couplings with a lower order coupling.

In our setup, there are two lower order couplings that correspond to a (partly) diffeomorphism invariant identification scheme, e.g. $\lambda _{5}$ can be identified with $\lambda _{3}$ or $\lambda _{4}$. In a well converged expansion scheme, the details of the identification should not matter and lead to similar results. In this section, we compare the results for different identification schemes in order to evaluate the stability of our expansion scheme.

The properties of the non-trivial UV fixed point for different identifications schemes are displayed in Table 2. In all identification schemes except for the identification $g_{n>4}\rightarrow g_{4}$ and $\lambda _{n>4}\rightarrow \lambda _{4}$ we find an attractive UV fixed point. In this case we can see that the fixed point has just vanished in the complex plane. For all other identifications we observe that the fixed point values and the critical exponents vary only mildly. Especially the number of attractive directions is consistently three with the first approximation to the stability matrix, c.f. Appendix 1. With the second approximation the number of attractive directions varies from one to three since the real part of one complex conjugated pair is close to zero.

In conclusion, this analysis suggests that our system is rather stable with respect to different identification schemes. Only one particular identification scheme has led to the disappearance of the attractive UV fixed point. This constitutes further support for our results in Sect. 5.2, where we found that our full system is very stable with respect to the identification of $g_{5}$ and $g_{6}$.

## Appendix D: Possible issues of a local momentum projection

In this section we want to point out some possible issues of a local momentum projection. A local momentum projection is for example a derivative expansion about a certain momentum, usually $p=0$.

The full solution of a flow equation includes a full resolution of the momentum dependence of all vertex flows. For higher *n*-point functions this task is computationally extremely challenging due to the high number of momentum variables. We have already argued in Sect. 4.2 that this task can be tremendously simplified with a symmetric momentum configuration. We have further shown in Sect. 4.3 that the quantity $\text {Flow}^{(n)}_G/(-\frac{n}{2} \eta _{h}(p^2) - n + 2)$ is polynomial in $p^2$, at least for $n=3,4$. Thus it is possible to consistently project on each coefficient of this polynomial in the whole momentum range $0\le p^2 \le k^2$ by employing a non-local momentum projection. In contrast, a local momentum projection scheme does not capture the correct momentum dependence over the whole momentum range $0\le p^2 \le k^2$ in general since it is sensitive to local momentum fluctuations. Furthermore, it is very challenging to project on the $p^4$ coefficient or even higher momentum order coefficients due to IR singularities. All these statements are explicitly exemplified in Fig. 6.

On the other hand, the local momentum projection at $p=0$ has the advantage that it allows for analytic flow equations, as discussed in Appendix 1. Analytic flow equations are more easily evaluated in the whole theory space, but, as the discussion above suggests, one should be be mindful of the fact that they easily introduce a large error.

We use the analytic flow equations in Sect. 6 precisely for the reason that they can easily be evaluated in the whole theory space. Thus we show now that the fixed point properties in this analytic system are qualitatively similar to the full system, despite the error that is introduced by the analytic equations.

The properties of the UV fixed point for different approximations are displayed in Table 3. Truncation 1 corresponds to our full system, i.e. with momentum dependent anomalous dimensions and bilocally evaluated gravitational couplings. Truncation 4 corresponds to the system used in Sect. 6, i.e. without anomalous dimensions and with gravitational couplings from a derivative expansion. Truncation 2 and 3 are in between those truncations, i.e. with anomalous dimension but gravitational couplings from a derivative expansion and without anomalous dimensions but with bilocally evaluated gravitational couplings, respectively.

We observe that the UV fixed point exists in all truncations, and that the properties of this fixed point vary only mildly. The fixed point values are all located within a small region, with the exception of our simplest truncation. There, the couplings $\lambda _{3}$ and $\lambda _{4}$ have a different sign compared to the other truncations. Considering the critical exponents we always find three attractive directions with the first approximation of the stability matrix and in three out of four cases three attractive directions with the second approximation of the stability matrix as well. These results suggest that it is an acceptable approximation to use the analytic flow equations if one is only interested in the qualitative behaviour of the system.

**Table 3:** Properties of the non-trivial UV fixed points for different approximations within the full system. In the truncations 3 and 4, we set $\eta _{\phi _i} = 0$, while in the truncations 1 and 2 we use momentum dependent anomalous dimensions. In truncations 2 and 4, the couplings $g_{3,4}$ are computed via a derivative expansion at $p=0$, while in the truncations 1 and 3 the couplings $g_{3,4}(k^2)$ are evaluated with a bilocal projection between $p=0$ and $p=k$. The quality of the truncation decreases from 1 to 4. The fixed point values are obtained with the identification scheme $\lambda _{n>4} = \lambda _{3}$ and $g_{n>4} = g_{4}$. The critical exponents ${\bar{\theta }}_i$ and $\tilde{\theta }_i$ stem from two different approximation of the stability matrix as discussed in Appendix 1. The fixed point properties from different approximations are qualitatively very similar. In particular, all fixed points exhibit three relevant directions when the first approximation of the stability matrix is used. Using the second approximation of the stability matrix also results in three relevant directions in three out of four cases

Trunc.	$\mu ^*$	$\lambda _{3}^*$	$\lambda _{4}^*$	$g_{3}^*$	$g_{4}^*$	${\bar{\theta }}_i$					${\tilde{\theta }}_i$				
1	$-\,0.45$	0.12	0.028	0.83	0.57	$-\,4.7$	$-\,2.0 \pm 3.1 \mathrm {i}$		2.9	8.0	$- \, 5.0$	$-\,0.37 \pm 2.4 \mathrm {i}$		5.6	7.9
2	$-\,0.41$	0.076	0.0055	0.71	0.53	$-\,4.0$	$-\,1.5 \pm 3.6 \mathrm {i}$		3.1	6.0	$- \, 3.9$	$-\,0.38 \pm 4.4 \mathrm {i}$		2.3	6.6
3	$-\,0.37$	0.049	0.0055	1.1	0.83	$-\,7.3$	$- \, 1.7 \pm 2.1 \mathrm {i}$		3.0	6.8	$- \, 7.2$	$0.32 \pm 2.7 \mathrm {i}$		4.7	6.8
4	$-\,0.23$	$-\,0.060$	$- \, 0.11$	0.64	0.55	$-\,3.0$	$- \, 1.9 \pm 1.6 \mathrm {i}$		1.7	3.4	$- \, 2.2$	$-\,0.50 \pm 1.7 \mathrm {i}$		$1.5 \pm 0.88 \mathrm {i}$	

## Appendix E: Derivation of flow equations

We obtain the flow equations for the individual coupling constants by projecting onto the flow of the graviton $n$-point functions, as explained in Sect. 4.5.

The equations for the graviton mass parameter $\mu$ and for the graviton anomalous dimension $\eta _{h}$ are extracted from the transverse-traceless part of the flow of the graviton two-point function. For $p^2 = 0$, we obtain

E1 $$\begin{aligned} \partial _t \mu = \left( \eta _{h}(0) - 2 \right) \mu + \frac{32 \pi }{5} \text {Flow}^{(hh)}_\mathrm{TT}(0), \end{aligned}$$

for the flow of the graviton mass parameter.

We obtain an equation for the graviton anomalous dimension $\eta _{h}(p^2)$ by evaluating the flow of the graviton two-point function bilocally at $p^2$ and $-\mu k^2$, to wit

E2 $$\begin{aligned} \eta _{h}(p^2)&= \frac{32 \pi }{5(p^2 + \mu k^2) } \left( \text {Flow}^{(hh)}_{\mathrm{TT}} \left( -\mu k^2 \right) \right. \nonumber \\&\quad -\left. \text {Flow}^{(hh)}_{\mathrm{TT}} \left( p^2 \right) \right) . \end{aligned}$$

The ghost anomalous dimension $\eta _{c}(p^2)$ can be directly obtained from the transverse flow of the ghost two-point function, to wit

E3 $$\begin{aligned} \eta _{c}(p^2)&= - \frac{ \text {Flow}^{(\bar{c}c)}_{\mathrm{T}} (p^2)}{3 p^2} . \end{aligned}$$

In case of the higher order couplings, we employ the projection operators described in Sect. 4.2. For the couplings $\lambda _{n}$, this leads to

E4 $$\begin{aligned} \partial _t \lambda _{n}&= \left( \frac{n}{2}\eta _{h}(0) +(n-4) -\frac{n-2}{2}\frac{\partial _t g_{n}}{g_{n}} \right) \lambda _{n} \nonumber \\&\phantom {=\,} + \frac{g_{n}^{1-\frac{n}{2}}}{C^{\Lambda _{n}}} \text {Flow}^{(n)}_{\Lambda _{n}}(0), \end{aligned}$$

where the projection dependent constant $C^{\Lambda _{n}}$ is implicitly defined via $\Pi _{\Lambda _n} \circ \Pi _\mathrm{TT}^n \circ \mathcal {T}^{(n)}(0;1) = C^{\Lambda _{n}}$. Here, $\circ$ denotes the pairwise contraction of indices.

As discussed in Sect. 4.5, the gravitational couplings $g_{n}(p^2)$ are momentum dependent. In order to simplify the computation we make an approximation of the full momentum dependence. This approximation exploits the fact that the flows are peaked at $p^2 = k^2$ and consequently we set the feed back on the right-hand side of the flow equation to $g_{n}(p^2) \approx g_{n}(k^2)$. This closes the flow equation for $g_{n}(k^2)$ and thus we only solve this equation. The easiest way to obtain the flow equation for $g_{n}(k^2)$ is a bilocal projection at $p=0$ and $p=k$. The resulting equation for $g_3(k^2)$ is precisely the same as in [3]. For $g_4(k^2)$ we obtain

E5 $$\begin{aligned} \partial _t g_{4}(k^2)&= 2 g_{4}(k^2) + 2 \eta _{h}(k^2)g_{4}(k^2) - C_4 g_{4}(k^2)\lambda _{4} (\eta _{h}(k^2)\nonumber \\&\quad - \eta _{h}(0)) + {C^{G_{4}}_{p^2}}^{-1}(\text {Flow}_G^{(4)}(k^2)- \text {Flow}_G^{(4)}(0))\,. \end{aligned}$$

The derivation of this equation is based on the assumption that $\lambda _{4}$ is small.

In Sect. 4.6 we have laid out a strategy to disentangle contributions from different tensor structures, in particular those of *R* and $R^2$. The flow equations for the $g_n$ are obtained by a projection onto the $p^2$ part of $\text {Flow}^{(n)}_G$ divided by $\left( -\frac{n}{2}\eta _{h}\left( p^2\right) - n + 2 \right)$, see Sect. 4.3 and Sect. 4.6. The graviton three-point function is at most quadratic in the external momentum, and consequently it is again enough to use a bilocal projection at $p^2 = 0$ and $p^2 = k^2$. Consequently, the flow equation for $g_3$ is quantitatively equivalent to the previous one if $\lambda _{3}$ is small. The graviton four-point function, on the other hand, has $p^4$ as its highest momentum power, and thus we use a trilocal momentum projection at $p^2=0$, $p^2=k^2/2$, and $p^2=k^2$. The flow equations of $g_{3}$ and $g_{4}$ are then given by

E6 $$\begin{aligned} \left( 1 + \eta _{3}\right) \partial _t g_{3}&= 2 g_{3} - 2 g_{3} C_3 \left( \partial _t \lambda _{3} +2 \lambda _{3}\right) \nonumber \\&\quad \left( \frac{1}{\frac{3}{2}\eta _{h}(k^2) + 1} - \frac{1}{\frac{3}{2}\eta _{h}(0) + 1} \right) + \frac{2}{C^{G_{3}}_{p^2}\sqrt{g_{3}}} \nonumber \\&\quad \left( \frac{\text {Flow}_G^{(3)}(k^2)}{\frac{3}{2}\eta _{h}(k^2) + 1} - \frac{\text {Flow}_G^{(3)}(0)}{\frac{3}{2}\eta _{h}(0) + 1} \right) , \end{aligned}$$

E7 $$\begin{aligned} \left( 1 + \eta _{4}\right) \partial _t g_{4}&= 2 g_{4} - g_{4} C_4 \left( \partial _t \lambda _{4} +2 \lambda _{4}\right) \left( - \frac{1}{\eta _{h}(k^2) + 1}\right. \nonumber \\&\quad \left. + \frac{4}{\eta _{h}(k^2/2) + 1} - \frac{3}{\eta _{h}(0) + 1} \right) \nonumber \\&\phantom {=\,} + \frac{1}{C^{G_{4}}_{p^2}}\left( - \frac{\text {Flow}_G^{(4)}(k^2)}{\eta _{h}(k^2) + 1} + 4 \frac{\text {Flow}_G^{(4)}(k^2/2)}{\eta _{h}(k^2/2) + 1}\right. \nonumber \\&\quad \left. - 3 \frac{\text {Flow}_G^{(4)}(0)}{\eta _{h}(0) + 1} \right) , \end{aligned}$$

where

$$\begin{aligned} \eta _{3}&= \frac{C_3 \lambda _{3} - \frac{3}{2}\eta _{h}(k^2)}{\frac{3}{2}\eta _{h}(k^2) + 1} - \frac{C_3 \lambda _{3}}{\frac{3}{2}\eta _{h}(0) + 1}, \nonumber \\ \eta _{4}&= \frac{-3 C_4 \lambda _{4}}{\eta _{h}(0) + 1} + 4\frac{C_4 \lambda _{4} -\frac{1}{2}\eta _{h}(k^2/2)}{\eta _{h}(k^2/2) + 1} - \frac{C_4 \lambda _{4} -\eta _{h}(k^2)}{\eta _{h}(k^2) + 1}. \end{aligned}$$

The constants *C* are implicitly defined via $\Pi _{G_n} \circ \Pi _\mathrm{TT}^n \circ \mathcal {T}^{(n)}(p^2;\Lambda _{n}) = C^{G_{n}}_{\Lambda _{n}} \Lambda _n +C^{G_{n}}_{p^2} p^2$, and we use the abbreviation $C_n :=C^{G_{n}}_{\Lambda _{n}}/C^{G_{n}}_{p^2}$. Again, $\circ$ denotes the pairwise contraction of indices. Note that the constants $\eta _{n}$ are chosen in such a way that $\eta _{n} = 0$ for vanishing anomalous dimensions.

Analogously, we can obtain a flow equation for the $R^2$ coupling of the graviton four-point function $\omega _4$ by using a trilocal momentum projection, as explained in Sect. 4.6. We evaluate the flows at the same momenta as for the trilocal flow equation of $g_{4}$. The equation for $\omega _4$ then reads

E8 $$\begin{aligned}&\left( 1 + \eta _{\omega } \right) \partial _t \omega _{4} = 2 \omega _{4} - \frac{\partial _t g_{4}}{g_{4}} \left( \frac{C^{G_{4}}_{\Lambda _{4}} \lambda _{4} + C^{G_{4}}_{p^2} + C^{G_{4}}_{\omega _{4}} \omega _{4}}{\eta _{h}(k^2) + 1} \right. \nonumber \\&\quad \left. - 2\frac{C^{G_{4}}_{\Lambda _{4}} \lambda _{4} + \frac{1}{2} C^{G_{4}}_{p^2} + \frac{1}{4} C^{G_{4}}_{\omega _{4}} \omega _{4}}{\eta _{h}(k^2/2) + 1} + \frac{C^{G_{4}}_{\Lambda _{4}} \lambda _{4}}{\eta _{h}(0) + 1} \right) \nonumber \\&\quad - \frac{C^{G_{4}}_{\Lambda _{4}}}{C^{G_{4}}_{\omega _{4}}} \left( \partial _t \lambda _{4} + 2\lambda _{4} \right) \left( \frac{1}{\eta _{h}(k^2) + 1}- \frac{2}{\eta _{h}(k^2/2) + 1}\right. \nonumber \\&\quad \left. + \frac{1}{\eta _{h}(0) + 1} \right) \nonumber \\&\quad + \frac{1}{C^{G_{4}}_{\omega _{4}} g_{4}} \left( \frac{\text {Flow}_G^{(4)}(k^2)}{\eta _{h}(k^2) {+} 1} {-} 2 \frac{\text {Flow}_G^{(4)}(k^2/2)}{\eta _{h}(k^2/2) {+} 1} {+} \frac{\text {Flow}_G^{(4)}(0)}{\eta _{h}(0) {+} 1} \right) , \end{aligned}$$

where

$$\begin{aligned} \eta _{\omega }&= \frac{\eta _{h}(k^2/2)}{\eta _{h}(k^2/2) + 1} - \frac{2\eta _{h}(k^2)}{\eta _{h}(k^2) + 1}. \end{aligned}$$

The constants *C* are again defined via the contraction $\Pi _{G_n} \circ \Pi _\mathrm{TT}^n \circ \mathcal {T}^{(n)}(p^2;\Lambda _{n}) = C^{G_{n}}_{\Lambda _{n}} \Lambda _n + C^{G_{n}}_{p^2} p^2+C^{G_{n}}_{\omega _{n}} \Omega _4 p^4$. Again, $\eta _{\omega }$ is defined such that $\eta _{\omega }=0$ for vanishing anomalous dimensions.

In the previous paragraphs we introduced abbreviations for constants that arise from the projection scheme. The explicit values of these constants are:

E9 $$\begin{aligned} C^{\Lambda _{3}}&= \frac{5}{192 \pi ^2},&C^{\Lambda _{4}}&= \frac{371881}{6718464\pi ^2}\,, \nonumber \\ C^{G_{3}}_{\Lambda _{3}}&= -\frac{9}{4096 \pi ^2},&C^{G_{4}}_{\Lambda _{4}}&= \frac{222485}{60466176\pi ^2}\,, \nonumber \\ C^{G_{3}}_{p^2}&= \frac{171}{32768 \pi ^2},&C^{G_{4}}_{p^2}&= \frac{6815761}{544195584\pi ^2}\,, \nonumber \\ C_3&= \frac{C^{G_{3}}_{\Lambda _{3}}}{C^{G_{3}}_{p^2}} = - \frac{8}{19}\,,&C_4&= \frac{C^{G_{4}}_{\Lambda _{4}}}{C^{G_{4}}_{p^2}} = \frac{2002365}{6815761}, \nonumber \\ C^{G_{4}}_{\omega _{4}}&= -\frac{96203921}{1632586752\pi ^2}.&\end{aligned}$$

If we want to obtain analytic flow equations for the gravitational couplings $g_{n}$, which are significantly less accurate, as discussed in Appendix 1, then we have to apply a partial derivative with respect to $p^2$ and evaluate the result at $p^2=0$. The resulting equations are given by

E10 $$\begin{aligned} \partial _t g_n&= 2 g_n+ \frac{n g_n}{n-2} \left( \eta _{h}(0) + C_n \lambda _n \eta _{h}'(0)\right) \nonumber \\&\quad +\frac{2}{n-2}\frac{g_{n}^{2-\frac{n}{2}}}{C^{G_{n}}_{p^2}} {\text {Flow}^{(n)}_{G}}'(0) \,, \end{aligned}$$

where $'$ denotes the dimensionless derivative with respect to $p^2$. These equations remain completely analytic if we use a Litim-shaped regulator [113] and approximate the anomalous dimensions as constant, $\eta _{\phi }\left( q^2\right) \approx \text {const.}$. We present the explicit analytic flow equations in the next Appendix.

## Appendix F: Analytic flow equations

All analytic flow equations are derived at $p^2 = 0$ (see e.g. (E10)) and with a Litim-shaped regulator [113]. The anomalous dimensions in the momentum integrals are approximated as constant, i.e. $\eta _{\phi _i} (q^2) \approx \eta _{\phi _i}$. It is usually a good approximation to use the anomalous dimensions evaluated at $k^2$ [71]. The analytic flow equations are then given by

F1 $$\begin{aligned} \partial _t \mu =&\left( \eta _{h}(0) - 2 \right) \mu \nonumber \\&+ \frac{1}{12 \pi }\frac{g_{4}}{(1+\mu )^2} \left( 3 (\eta _{h} - 8) - 8 \lambda _{4} (\eta _{h}-6) \right) \nonumber \\&- \frac{1}{180 \pi }\frac{{g_{3}}}{(1+\mu )^3} \left( 21 (\eta _{h} - 10) \right. \nonumber \\&\left. - 120 \lambda _{3} (\eta _{h} - 8) +320 \lambda _{3}^2 (\eta _{h} - 6) \right) \nonumber \\&+ \frac{g_{3}}{5 \pi } (\eta _{c} - 10), \end{aligned}$$

F2 $$\begin{aligned} \partial _t \lambda _3 =&\left( \frac{3}{2} \eta _{h}(0) - 1 -\frac{1}{2} \frac{\partial _t g_3}{g_{3}}\right) \lambda _{3} \nonumber \\&- \frac{1}{8 \pi }\frac{{g_{3}}^{-\frac{1}{2}} {g_{5}}^{\frac{3}{2}}}{(1+\mu )^2} \left( (\eta _{h} - 8) - 4 \lambda _{5} (\eta _{h}-6) \right) \nonumber \\&- \frac{1}{6 \pi }\frac{{g_{4}}}{(1+\mu )^3} \left( 3 \lambda _{4} (\eta _{h} - 8) - 16 \lambda _{3} \lambda _{4} (\eta _{h} - 6) \right) \nonumber \\&+ \frac{1}{240 \pi }\frac{ {g_{3}}}{(1+\mu )^4} \left( 11 (\eta _{h} - 12) - 72 \lambda _{3} (\eta _{h} - 10) \right. \nonumber \\&\left. + 120 {\lambda _{3}}^2 (\eta _{h} - 8) - 80 {\lambda _{3}}^3 (\eta _{h} - 6) \right) \nonumber \\&- \frac{g_{3}}{10 \pi } (\eta _{c} - 12), \end{aligned}$$

F3 $$\begin{aligned} \partial _t \lambda _4 =&\left( 2 \eta _{h}(0) - \frac{\partial _t g_4}{g_{4}}\right) \lambda _{4} + \frac{1}{13387716\pi } \nonumber \\&\phantom {+\cdot \,} \Bigg (\frac{1}{2}\frac{{g_{6}}^2 {g_{4}}^{-1}}{(1+\mu )^2} \left( -4472787 (\eta _{h} - 8) \right. \nonumber \\&\left. +\, 1639004 \lambda _{6} (\eta _{h}-6) \right) \nonumber \\&+ \frac{1}{15}\frac{{g_{4}}}{(1+\mu )^3} \left( 5066361 (\eta _{h} - 10)\right. \nonumber \\&\left. -\, 22517160 \lambda _{4} (\eta _{h} - 8) + 283174360 {\lambda _{4}}^2 (\eta _{h} - 6) \right) \nonumber \\&+ \frac{2}{15} \frac{{g_{3}}^{\frac{1}{2}} {g_{5}}^{\frac{3}{2}} {g_{4}}^{-1}}{(1{+}\mu )^3}\! \left( 3940503 (\eta _{h} {-} 10) {-} 60 ( 187643 \lambda _{3}\right. \nonumber \\&\left. - 1303286 \lambda _{5})(\eta _{h} - 8) + 417051520 \lambda _{3} \lambda _{5} (\eta _{h} - 6) \right) \nonumber \\&+ \frac{2}{5} \frac{g_{3}}{(1+\mu )^4} \left( - 1313501 (\eta _{h} - 12) \right. \nonumber \\&\left. +\, 3377574 (2 \lambda _{3} + \lambda _{4}) (\eta _{h} - 10) \nonumber \right. \nonumber \\&\left. -\, 15011440 (\lambda _{3} + 2 \lambda _{4}) \lambda _{3} (\eta _{h} - 8)\right. \nonumber \\&\left. +\, 45442920 {\lambda _{3}}^2 \lambda _{4} (\eta _{h} - 6) \phantom {\frac{1}{2}\frac{{g_{6}}^2 {g_{4}}^{-1}}{(1+\mu )^2}}\right) \nonumber \\&+ \frac{1}{5}\frac{ {g_{3}}^2 {g_{4}}^{-1} }{(1+\mu )^5} \bigg ( 2874147 (\eta _{h} - 14) \nonumber \\&- 20879816 \lambda _{3} (\eta _{h} - 12) + 36027456 {\lambda _{3}}^2 (\eta _{h} - 10) \nonumber \\&\phantom {+\cdot \,} + 88161840 {\lambda _{3}}^3 (\eta _{h} {-} 8) {-}248160672 {\lambda _{3}}^4 (\eta _{h} {-} 6) \bigg )\nonumber \\&- \frac{10426288}{7} \frac{{g_{3}}^2}{g_{4}}(\eta _{c} - 14) \Bigg ), \end{aligned}$$

F4 $$\begin{aligned} \partial _t g_{3} =&\left( 2 + 3 \eta _{h}(0) - \frac{8}{19} \eta _{h}'(0) \lambda _{3} \right) g_{3} + \frac{1}{19 \pi } \nonumber \\&\times \Big (\frac{{g_{3}}^{\frac{1}{2}} {g_{5}}^{\frac{3}{2}}}{(1+\mu )^2} \frac{47}{6} (\eta _{h} - 6) \nonumber \\&+ \frac{g_{3} g_{4}}{18(1+\mu )^3} \left( - 45 (\eta _{h} - 8) \right. \nonumber \\&+ 8 \left( 30 \lambda _{3} - 59 \lambda _{4} \right) (\eta _{h}-6)\nonumber \\&\left. + 360 \lambda _{3} \lambda _{4} (\eta _{h}-4) \right) + \frac{g_{3} g_{4}}{(1+\mu )^4} 16 \left( 1 - 3 \lambda _{3} \right) \lambda _{4} \nonumber \\&- \frac{{g_{3}}^2}{80(1+\mu )^4} \left( 147 (\eta _{h}-10) - 1860 \lambda _{3} (\eta _{h}-8) \right. \nonumber \\&\left. +\, 3380 \lambda _{3}^2 (\eta _{h}-6) + 25920 \lambda _{3}^3 (\eta _{h}-4) \right) \nonumber \\&- \frac{2 {g_{3}}^2}{15(1+\mu )^5} \left( 229 - 1780 \lambda _{3} + 3640 \lambda _{3}^2 - 2336 \lambda _{3}^3 \right) \nonumber \\&+ \frac{{g_{3}}^{2}}{10} \left( 53 (\eta _{c}-10) + 480 \right) \Big ), \end{aligned}$$

F5 $$\begin{aligned} \partial _t g_{4} =&2 \left( 1 + \eta _{h}(0) + \frac{2002365}{6815761} \eta _{h}'(0) \lambda _{4} \right) g_{4} + \frac{2125764}{6815761 \pi } \nonumber \\&\times \Bigg (\frac{{g_{6}}^{2}}{(1+\mu )^2} \frac{32830375}{25509168} (\eta _{h} - 6) \nonumber \\&-\frac{{g_{4}}^2}{76527504(1+\mu )^3 } \left( 11305705 (\eta _{h} - 8) \right. \nonumber \\&\left. +\, 61298276 \lambda _{4} (\eta _{h}-6) + 308793960 \lambda _{4}^2 (\eta _{h}-4) \right) \nonumber \\&-\frac{4 {g_{4}}^2}{3188646(1+\mu )^4} \left( 16061481 + 8 \left( 5355213 \lambda _{4} \right. \right. \nonumber \\&\left. \left. -\,5610604\right) \lambda _{4} \right) \nonumber \\&-\frac{{g_{3}}^{\frac{1}{2}} {g_{5}}^{\frac{3}{2}}}{19131876(1+\mu )^3} \Big (-34242339 (\eta _{h} - 8) \nonumber \\&+ \left( 86256922\lambda _{3} - 7511302\lambda _{5}\right) (\eta _{h}-6)\nonumber \\&-4483422 \lambda _{3}\lambda _{5} (\eta _{h}-4) \Big ) \nonumber \\&- \frac{{g_{3}}^{\frac{1}{2}} {g_{5}}^{\frac{3}{2}}}{(1+\mu )^4} \left( 784609\left( 17-32\lambda _{3}\right) \right. \nonumber \\&\left. -\, 8937232 (4-9 \lambda _{3}) \lambda _{5} \right) \nonumber \\&+\frac{{g_{3}} {g_{4}}}{90(1+\mu )^4} \Big (323831781 (\eta _{h} - 10)\nonumber \\&-\left( 80\cdot 11179796\lambda _{3} + 203187860\lambda _{4}\right) (\eta _{h}-8) \nonumber \\&-\left( 10\cdot 129631943\lambda _{3} - 80\right. \nonumber \\&\left. \cdot 16941407\lambda _{4}\right) \lambda _{3} (\eta _{h}-6)\nonumber \\&-10\cdot 292902984 \lambda _{3}^2\lambda _{4}(\eta _{h}-4)\Big ) \nonumber \\&+ \frac{{g_{3}} {g_{4}}}{15(1+\mu )^5} \Big (26769135 \left( 17+8 \lambda _{3} \left( 9 \lambda _{3} - 8\right) \right) \nonumber \\&-2 \left( 353519805 + 4 \lambda _{3} \left( 742510961 \lambda _{3} \right. \right. \nonumber \\&\left. \left. -\,514449355\right) \right) \lambda _{4}\Big ) \nonumber \\&+\frac{2{g_{3}} {g_{4}}}{9(1+\mu )^4} \Big (6783386859 (\eta _{h} - 10)\nonumber \\&-\left( 140\cdot 86837935\lambda _{3} + 457106270\lambda _{4}\right) (\eta _{h}-8) \nonumber \\&-\left( 20\cdot 4067950507\lambda _{3} + 140\right. \nonumber \\&\left. \cdot 799593508\lambda _{4}\right) \lambda _{3} (\eta _{h}-6) -20\nonumber \\&\cdot 25136284404 \lambda _{3}^2\lambda _{4}(\eta _{h}-4) \Big ) \nonumber \\&+ \frac{{g_{3}} {g_{4}}}{15(1+\mu )^5} \Big (394709295 - 661068650 \lambda _{4} \nonumber \\&+ 40 \left( 91735671 \lambda _{4} -34781816\right) \lambda _{3} \nonumber \\&- 8 \left( 731880777 \lambda _{4} -220800215\right) \lambda _{3}^2\Big ) \nonumber \\&+\frac{{g_{3}}^2}{45(1+\mu )^5} \Big (-125220803(\eta _{h}-12) +2\nonumber \\&\cdot 284391180\lambda _{3}(\eta _{h}-10) \nonumber \\&+2\cdot 167456175\lambda _{3}^2(\eta _{h}-8) -2\cdot 236\nonumber \\&\cdot 13640606\lambda _{3}^3(\eta _{h}-6) + 2\cdot 236\nonumber \\&\cdot 36717495 \lambda _{3}^4(\eta _{h}-4) \Big ) \nonumber \\&- \frac{{g_{3}}^2}{3(1+\mu )^6} \Big (112533531 -855576992\lambda _{3} \nonumber \\&+3683259968\lambda _{3}^2\nonumber \\&-7947008128\lambda _{3}^3 +6385327072\lambda _{3}^4\Big )\nonumber \\&+4 {g_{3}}^{2} \left( \frac{23005837}{5} (\eta _{c}-12) + 11171540 \right) \Bigg ). \end{aligned}$$
